# Supplementary material for: Absence of fibroin H sequences and a significant divergence in the putative fibroin L homolog in Neomicropteryx cornuta (Micropterigidae) silk
Source: Commun Biol. 2025 Mar 13;8:434. doi: 10.1038/s42003-025-07801-w (PMC11906653; doi:10.1038/s42003-025-07801-w)
Supplement: Supplementary file 2 — Supplementary Information [file 42003_2025_7801_MOESM2_ESM.pdf]

Supplementary Figure 1: Amino acid sequence alignment of putative FibL proteins. The alignment was generated with the ClustalW Multiple Sequence Alignment in BioEdit (1). Residues are color-coded according to their physicochemical properties. Notably, the sequence from *N. cornuta* displays significant divergence compared to the other aligned sequences. *P. californicus* (GU180674); *E. semipurpurella* (PQ040460); *T. bisselliella* (MW244681); *G. mellonella* (XM_0268957); *B. mori* (NM_001044023); *L. lunatus* (BK062791); *H. angustipennis* (AB354593); *R. obliterate* (AB354590); *P. conspersa* (OL589402); *N. cornuta* (BankIt2830184).

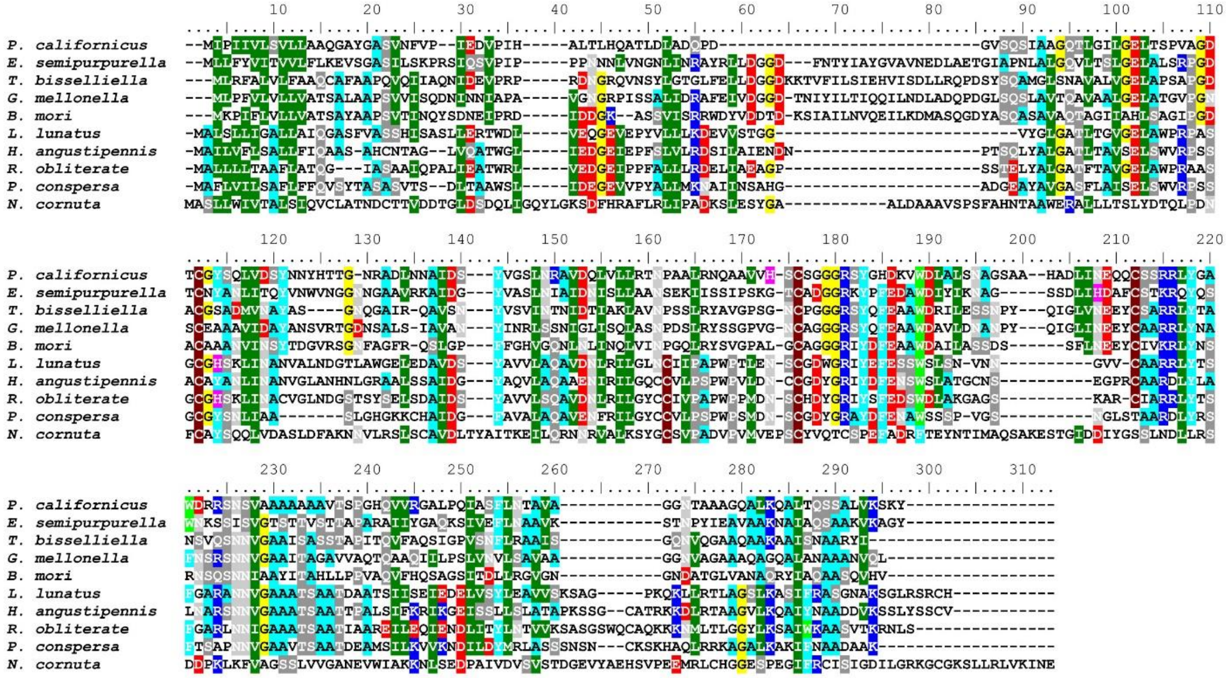

Supplementary Figure 2: Phylogenetic trees of FibL. The sequences of moths and caddisflies are shown together with the related sequence FibX of *N. cornuta*. A) The phylogram was created in MEGA (2) using the neighbor-joing method (p-distance, bootstrap with 1000 replicates), B) the phylogram was created with the online version of IQ-Tree (3) inferred using the maximum likelihood method (TVM+F+R2, ultra-fast bootstrap with 1000 replicates) – the phylogram was rooted with the trichopteran group. The accession numbers of the FibL proteins are listed in the legend in Figure S1.

A

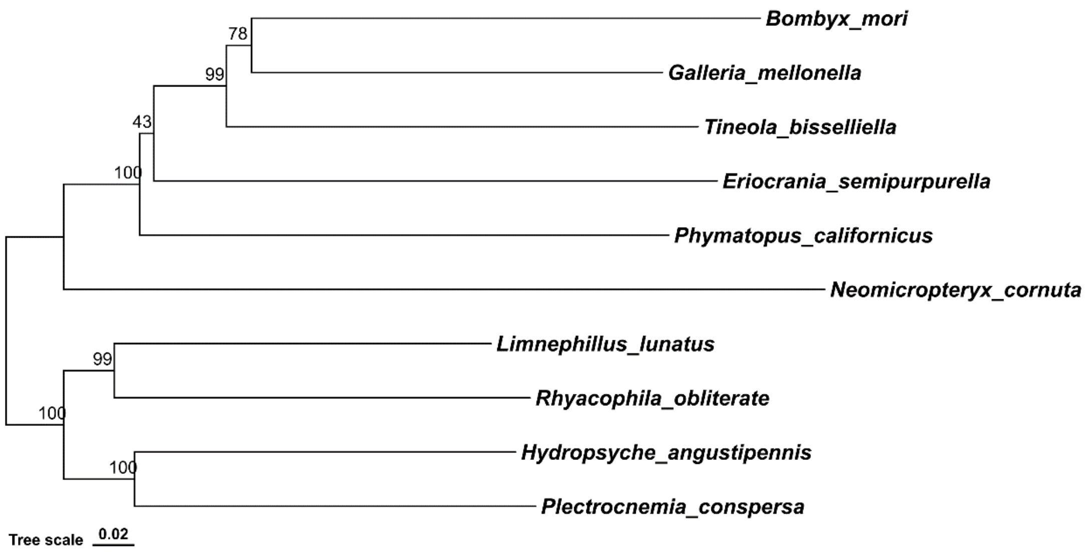

B

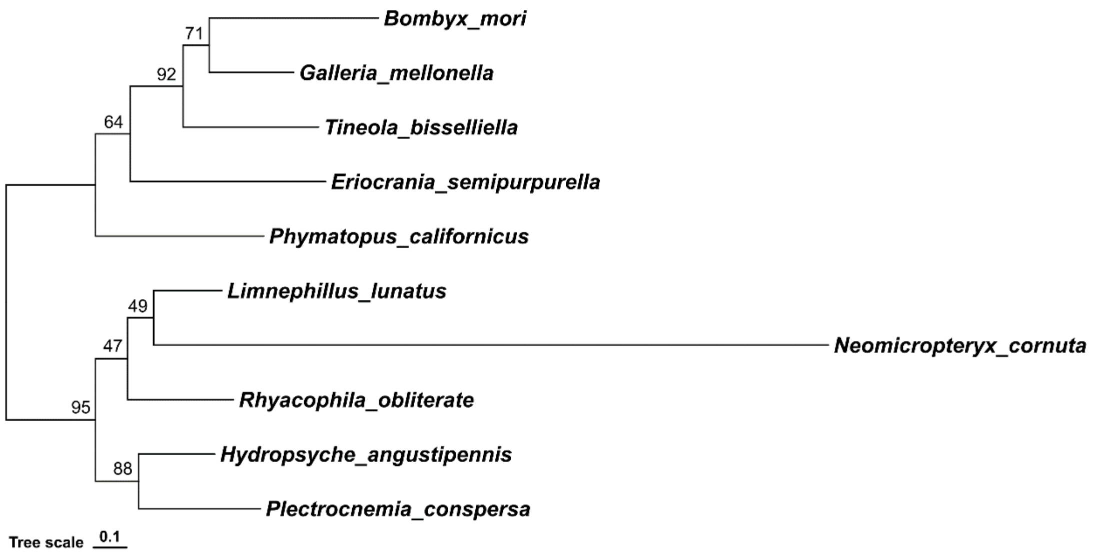

Supplementary Figure 3: Head of *N. cornuta.* A) Full head view. B) The detailed view of the opening of the labial salivary gland (arrowhead) with spinning silk thread (arrow). Asterisks show scutate cuticular outgrowths from the head-prothoracic fold: *an*, antenna; *lb*, labrum; *M*, medial seta; *md*, mandibula, *mx*, maxilla; *st*, stemmata. Scale bars: 10 µm.

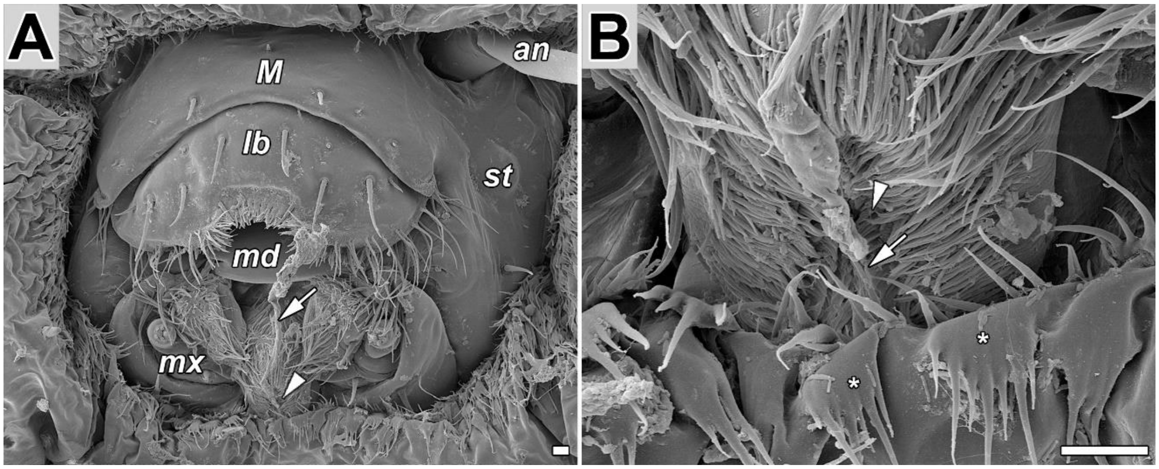

Supplementary Figure 4: Amino acid sequences of major putative silk structural proteins from *Neomicroteryx cornuta* listed in Table 1. Signal peptides important for secretion are shown in red. Some of the repeated sequence motifs within the proteins are highlighted to illustrate their complexity and potential functional significance for silk fiber formation.

A) Sericin-like 1 (PV091728)

MKVLALFFVAFAVILNIKATDAKPQICVKKVKTSDEKPGSNGFWGMFAAEPKDEEFCYDPDTPPKSSELKLLGYTGEKYNKSNSVQSSS
SSSTSKSSTSSTKPSPNPGKLIVTTVETTTTTVNGVTTKSHHTTTKEIDDTDAGCKSAEVSTLPAKLQRSKRDTSETLAGLENEKFASS
GPITDKQCLCNTKITQISCIHCSPQGNRYAIEKSCRSEDGVVGPVKVSYVNDLKDILGTDDKLNKHNSGDCDSSSSSSSSSDSSSSSDS
SSSSSSESSSSSSSDSSSSSDSSESSSSSEEEDDKVIGKSSKSQVKSISASKTSQLDIHKGSYKTKSDSLKSGSKKGGKDDSSSSSSS
SSSSSSSSDSSSSSSSSESSEEDDIKGGGAEATAATQSTIQVGAHKSIAAPKSIKLKEEIKCA

B) Sericin-like 2 (PV091729)

MRAFSPFGLLFTGICLAIALNCGGSDANADWLSNIEDGEENKEVKQSVKPNWSDVSRESRGNNEWFKQGNVNKSCKKAEKHEKQQRLLD
QVETIIEELRPKTIWQKAIQKKTSNSESSQSNFVSCKSSNTNSKSNTETKSTSGTKTISKSKALSNAKKAKILRKSIASKKCCSKSKKG
TKLRKTYKSKSSSYSSSSSKSVSSSSSGSKSGSSASSGSKSGSSSSGSKSGSSSSRGSRAGSSASSGSKSGSSSSSGSRAGSSASSVSK
SGSSASSGSKSCSSASSGSKSGSSASSGSKSCSSASSGSKSSSSASTGSKSGSSSSSGSRAGSSASSGSKSSSSASSGSKSSSSASTGS
KSGSSSSSGSRAGSSASSGSKSGSSASSASKSGISSSSGSRAGSSASSGSKSGSSASSGSKSSSSASTGSKSGSSSSSGSRAGSSASSG
SKSSSSASSGSKSSSSASTGSKSGSSSSGSRAGSSASSGSKSGSSASSGSKSSSSASSGSKSSSSASSGSKSSSSASTGSKSGSSSSS
GSRAGSSASSGSKSGSSASSASKSGISSSSGSRAGSSASIGSKSGVSSSSGSRVGSSASSGSKSGSSASSGSKSCSSASSGSKSSSSAS
TGSKSGSSSSSGSRAGSSASSGSKSSSSASSGSKSSSSASTGSKSGSSSSGSRAGSSASSGSKSGSSASSGSKSGSSASSASKSGISS
SSGSRAGSSASIGSKSGVSSSSGSRAGSSASSGSKSSSSASSGSKSSSSASTGSKSGSSSSSGSRAGSSASSGSKSGSSASSASKSGIS
SSSGSRAGSSASSVSKSSGSKSGSSASSVSKSGSSASSGSKSGSSASSGSKSGSSSSSGSRAGSSASSGSKSGSSASSASKSCISSSSG
SRAGSSASSVSKSSGSRAGSSASSVSKSGSSASSGSKSSSSASSGSKSGSSASSGSKSGSSASSGSKSCSSASSGSKSSSSASTGSKSG
SSSSSGSRAGSSASSGSKSSSSASSGSKSSSSASTGLKSGSSSSSGSRAGSSASSGSKSGSSASSGSKSGSSASSGSKSGSSASSGSKS
CSSASSGSKSSSSASTGSKSGSSSSSGSRAGSSASSGSKSSSSASSGSKSSSSASTGSKSGSSSSGSRAGSSASSVSKSGSSASSGSK
SGSSASSGSKSGSSASSGSKSCSSASSGSKSSSSASTGSKSGSSSSSGSRAGSSASSGSKSSSSASSGSKSSSSASTGLKSGSSSSSGS
RAGSSASSGSKSGRSASSASKSGISSSSGSRAGSSASSVSKSSGSRAGSSASSVSKSGSSASSGSKSGSSASSGSKSGSSSSSGSRAGS
SASSGSKSGSSASIGSKSGVSSSSGSRAGSSASSVSKSSSSASSGSKSSSSASTGLKSGSSSSSGSRAGSSASSGSKSGSSASSGSKSS
SSASTGSKSGSSSSSGSRAGSSASIGSKSGVSSSSGSRAGSSASSGSKSSSSASTASKSGISSSSGSRAGSSASSGSKSSSSASSGSKS
SSSASTGSKSGSSSSSGSRAGSSASSGSKSGRSASSASKSGISSSSGSRAGSSASSVSKSSGSRAGSSASSVSKSGSSASSGSKSSSSA
STGSKSGSSSSSGSRAGSSASSGSKSGRSASSASKSGISSSSGSRAGSSASSVSKSSGSRAGSSASSVSKSGSSASSGSKSGSSASSGS
KSGSSSSSGSRAGSSASSGSKSGSSASSGSKSGSSASIGSKSGVSSSSGSRAGSSASSVSKSSSSASSGSKSSSSASTGLKSGSSSSSR
SRAGSSASSGSKSGSSASSGSKSSSSASTGSKSGSSSSSGSRAGSSASTGSKSGSSSSSGSRAGSSASSGSKSGSSASSASKSGISSSS
GSRAGSSASSVSKSSGSRAGSSASSVSKSGSSASSGSKSGSSASSGSKSGSSSSSGSRAGSSASSVSKSSGSRAGSSASSVSKSGSSAS
SGSKSGSSASSGSKSGSSSSSGSRAGSSASSVSKSGSSASSGSKSCSSASTGSKSGSSSSSGSRAGSSASSGSKSSSSASSGSKSGSSS
SSGSRAGSSASSGSKSSSSASSGSKSSSSASTGLKSGSSSSSGSRAGSSASSGSKSGSSASSASKSGISSSSGSRAGSSASIGSKSGVS
SSSGSRAGSSASSGSKSSSSASSGSKSSSSASTGSKSGSSSSSGSRAGSSASSGSKSGRSASSASKSGISSSSGSRAGSSASSVSKSSG
SRAGSSASSVSKSGSSASSGSKSGSSASSGSKSGSSASSGSKSCSSASSGSKSGSSASSGSKSSSASTGSKSGSSSSSGSRAGSSASS
GSKSSSSASSGSKSSSSASTGSKSGSSSSGSRAGSSASSGSKSGSSASSGSKSGSSSSSGSRVGSSASSGSKSGSSASSGSKSCSSAS
SGSKSSSSASTGSKSGSSSSSGSRAGSSASSVSKSSGSRAGSSASSVSKSGSSASSGSKSGSSSSSGSRAGSSASSGSKSCSSASSGSK
SGSSASTGSKSGSSSSSGSRAGSSASSGSKSSSSASSGSKSSSSASTGSKSGSSSNGSRAGSSASSGSKSGSSASSGSKSGSSSSSGS
RAGSSASIGSKSGVSSSSGSRVGSSASSGSKSGSSASSGSKSCSSASSGSKSSSASTGSKSGSSSSSGSRAGSSASSGSKSSSSASSG
SKSSSSASTGSKSGSSSSGSRAGSSASSGSKSGSSASSASKSGISSSSGSRAGSSASIGSKSGVSSSSGSRAGSSASSVSKSSSSASS
GSKSSSSASTGLKSGSSSSSRSRAGSSASSGSKSGSSASSGSKSSSSASTGSKSGSSSSSGSRAGSSASTGSKSGSSSSSGSRAGSSAS
SGSKSGSSASSASKSGISSSSGSRAGSSASSVSKSSGSRAGSSASSVSKSGSSASSGSKSGSSASSGSKSGSSSSSGSRAGSSASSVSK
SSGSRAGSSASSVSKSGSSASSGSKSGSSASSGSKSGSSSSSGSRAGSSASSVSKSGSSASSGSKSCSSASTGSKSGSSSSSGSRAGSS
ASSGSKSSSSASSGSKSGSSSSSGSRAGSSASSGSKSSSSASSGSKSSSSASTGLKSGSSSSSGSRAGSSASSGSKSGSSASSASKSGI
SSSSGSRAGSSASIGSKSGVSSSSGSRAGSSASSGSKSSSSASSGSKSSSSASTGSKSGSSSSSGSRAGSSASSGSKSGRSASSASKSG
ISSSSGSRAGSSASSVSKSSGSRAGSSASSVSKSGSSASSGSKSGSSASSGSKSGSSASSGSKSCSSASSGSKSGSSASSGSKSSSAS
TGSKSGSSSSSGSRAGSSASSGSKSSSSASSGSKSSSSASTGSKSGSSSSGSRAGSSASSGSKSGSSASSGSKSGSSSSSGSRVGSSA
SSGSKSGSSASSGSKSCSSASSGSKSSSASTGSKSGSSSSSGSRAGSSASSGSKSSSSASSGSKSSSSASTGSKSGSSSSGSRAGSS
ASSGSKSGSSASSASKSGISSSSGSRAGSSASSVSKSSGSRAGSSASSVSKSGSSASSGSKSGSSASSGSKSSSSASTGSKSCSSASSG
SKSSSSASTGSKSGSSSSSGSRAGSSASSGSKSGSSASSASKSGISSSSGSRAGSSASSVSKSSGSRAGSSASSVSKSGSSASSGSKSG
SSASSGSKSGSYSSSGSRAGSSASSVSKSSGSRAGSSASSVSKSGSSASSGSKSGSSASSGSKSGSSSSSGSRAGSSASSVSKSGSSAS
SVSKSGVSSSSGSRVGSSASSGSKSGSSSSSGSRAGSSASSGSKSGSSASSGSKSSSASTGSKSGSSSSSGSRAGSSASSGSKSSSSA
SSGSKSSSSASTGSKSGSSSNGSRAGSSASSGSKSGSSASSGSKSGSSSSSGSRAGSSASIGSKSGVSSSSGSRVGSSASSGSKSGSS
ASSGSKSCSSASSGSKSSSASTGSKSGSSSSSGSRAGSSASSGSKSSSSASSGSKSSSSASTGSKSGSSSSGSRAGSSASSGSKSGS
SASSASKSGISSSSGSRAGSSASSVSKSSGSRAGSSASSVSKSGSSASSGSKSGSSASSGSKSGSSASSGSKSGSSSSSGSRAGSSASS
GSKSGSSASSASKSGISSSSGSRAGSSASSVSKSSGSRAGSSASSVSKLGSSASSGSKSGSSASSGSKSGSSASSGSKSGSSASSGSKS
GSYSSSGSRAGSSASSVSKSGSSASSGSKSCSSATSGSKSGSSASSGSKSCSSAFSGLKSSSSASTGSKSGSSSSSGSRAGSSASSGSK
SSSSASSGSKSSSSASTGSKSGSSSSGSRAGSSASSGSKSGSSASSASKSGSSASSGSKSCSSASSGSKSSSSASTGSKSGSSSSSGS
RAGSSASSGLKSGSSASSASKSGISSSSGSRAGSSASSVSKSSGSRAGSSASSVSKSGSSASSGSKSGSSASSGSKSGSSSSSGSRAGS
SASSVSKSSGSRAGSSASSVSKSGSSASSGSKSGSSASSGSKSGSSSSSGSRAGSSASSVSKSGSSASSVSKSGVSSSSGSRVGSSASS
GSKSGSSSSSGSRAGSSASSVSKSSGSRAGSSASSVSKSGSSASSGSKSCSSASSGSKSGSSASSGSKSSSSASTGSKSGSSSSSGSRA

GSSASSVSKSSSSASSGSKSSSSASTGSKSGSSSSSGSRAGSSASSGSKSGSSASSASKSGISSSSGSRAGSSASSVSKSSGSRAGSSA
SSVSKSGSSASSGSKSGSSASSGSKSGSYSSSGSRAGSSASSVSKSSGSRAGSSASSVSKSGSSASSGSKSGSSASSGSKSGSSSSSGS
RAGSSASSVSKSGSSSSSGSRAGSSASSVSMSGSSASSGSKSGSSSSSGSRAGSSASSGLKSGSSASSGSKSRSSASSGSKSSSSASTG
SKSGSSSSSGSRAGSSASSGSKSSSSASSDSKSSSSASSGSKSSSSASTGSKSGSSSSSGSRAGSSASSGSKSSSSASSGSKSSSSAST
GSKSGSSSSSGSRAGSSASSVSKSSGSRAGSSASSVSKSGSSASSGSKSGSSASSGSKSCSSASSGSKSSSSASTGSKSGSSSSSGSRA
GSKSSSSASTGSKSGSSSSSRSRAGSSASSGSKSGSSASSASKSGISSSSGSRAGSSASIGSKSGVSSSSGSRVGSSASSGSKSGSSAF
SGSKSCSSASSVSKSSSSASTGSKSGSSSSSGSRAGSSASSGSKSSSSASSGSKSSSSASTGSKSGSSSSSGSRVGSSASSGSKSGSSA
SSASKSCISSSSGSRAGSSASSVSKSSGSRAGSSASSVSKSGSSASSGSKSGSSASSGSKSGSSSSSGSRAGSSASSVSKSSGSRAGSS
ASSVSKSGSSASSGSKSGSSASSGSKSGSSSSSGSRAGSSASSVSKSGSSASSGSKSGSSASSGSKSCSSASSGSKSSSSASTGSKSGS
SSSSGSRAGSKSSSSASTGSKSGSSSSSRSRAGSSASSGSKSGSSASSASKSGISSSSGSRAGSSASIGSKSGVSSSSGSRVGSSASSG
SKSGSSASSGSKSCSSASSVSKSSSSASTGSKSGSSSSSGSRAGSSASSGSKSSSSASSGSKSSSSASTGSKSGSSSSGSRVGSSASS
GSKSGSSASSASKSCISSSSGSRAGSSASSVSKSSGSRAGSSASSVSKSGSSASSGSKSGSSASSGSKSGSSSSSGSRAGSSASSVSKS
SGSRAGSSASSVSKSGSSASSGSKSGSSASSGSKSGSSSSSGSRAGSSASSVSKSGSSASSVSKSGVSSSSGSRVGSSASSGSKSGSSS
SSGSRAGSSASSVSKSSGSRAGSSASSVSKSGSSASSGSKSCSSASSGSKSGSSASSGSKSCSSASSGSKSSSSASTGSKSGSSSSSGS
RAGSSASSGSKSGSSASSGSKSGSSASSGSKSGSSSSSGSRAGSSASSVSKSGSSASSVSKSGVSSFSGSRVGSSASSGSKSGSSSSSG
SRAGSSASSGSKSGSSASSGSKSRSSASSGSKSSSSASTGSKSGSSSSSGSRAGSSASSGSKSSSSASSGSKSSSSASSGSKSSSSAST
GSKSGSSSSSGSRAGSSASSGSKSSSSASSGSKSSSSASTGSKSCSSASSGSKSSSSASTGSKSGSSSSSGSRAGSSASSGSKSGSSAS
SASKSGISSSSGSRAGSSASSVSKSSGSRAGSSASSVSKSGSSASSGSKSGSSASSGSKSGSYSSSGSRAGSSASSVSKSSGSRAGSSA
SSVSKSGSSASSGSKSGSSASSGSKSGSSSSSGSRAGSSASSVSKSGSSASSVSKSGVSSSSGSRVGSSASSGSKSGSSSSSGSRAGSS
ASSGSKSGSSASSGSKSGSSASTDSKSGSSSSSGSRAGSSASSGSKSSSSASSGSKSSSSASTGSKSGSSSSSGSRAGSSASSGSKSGS
SASSGSKSGSSSSSGSRAGSSASIGSKSGVSSSSGSRVGSSASSGSKSGSSASSGSKSCSSASSGSKSSSSASTGSKSGSSSSSGSRAG
SSASSGSKSSSSASSGSKSSSSASTGSKSGSSSSSGSRAGSSASSGSKSGSSASSASKSGISSSSGSRAGSSASSVSKSSGSRAGSSAS
SVSKSGSSASSGSKSGSSASSGSKSGSSASSGSKSGSSSSSGSRAGSSASSGSKSGSSASSASKSGISSSSGSRAGSSASSVSKSSGSR
AGSSASSVSKLGSSASSGSKSGSSASSGSKSGSSSSSGSRAGSSASSVSKSSGSRAGSSASSVSKSGSSASSGSKSGSSASSGSKSGSS
SSSGSRAGSSASSGSKSGSSASSGSKSCSSASSGSKSSSSASTGSKSGSSSSSGSRAGSSASSGSKSGSSASSGSKSGSSASSGSKSGS
SSSSGSRAGSSASSVSKSGSSASSVSKSGVSSFSGSRVGSSASSGSKSGSSSSSGSRAGSSASSGSKSSGSRAGSSASSGSKSGSSASS
GSKSCSSASSGSKSGSSASSGSKSCSSASSGSKSSSSASTGSKSGSSSSSGSRAGSSASSGSKSGSSASSGSKSGSSASSGSKSGSSSS
SGSRAGSSASSVSKSGSSASSVSKSGVSSFSGSRVGSSASSGSKSGSSSSSGSRAGSSASSGSKSGSSASSGSKSRSSASSGSKSSSSA
STGSKSGSSSSSGSRAGSSASSGSKSSSSASSGSKSSSSASSGSKSSSSASTGSKSGSSSSSGSRAGSSASSGSKSSSSASSGSKSSSS
ASTGSKSCSSASSGSKSSSSASTGSKSGSSSSSGSRAGSSASSGSKSGSSASSASKSGISSSSGSRAGSSASSVSKSSGSRAGSSASSV
SKSGSSASSGSKSGSSASSGSKSGSYSSSGSRAGSSASSVSKSSGSRAGSSASSVSKSGSSASSGSKSGSSASSGSKSGSSSSSGSRAG
SSASSVSKSGSSASSVSKSGVSSSSGSRVGSSASSGSKSGSSSSSGSRAGSSASSGSKSGSSASSGSKSCSSASSGSKSSSSASTGSKS
GSSSSSGSRAGSSASSGSKSGSSASSGSKSGSSASSGSKSGSSSSSGSRAGSSASSVSKSGSSASSVSKSGVSSFSGSRVGSSASSGSK
SGSSSSSGSRAGSSASSGSKSGSSASSGSKSRSSASSGSKSSSSASTGSKSGSSSSSGSRAGSSASSGSKSSSSASSGSKSSSSASSGS
KSSSSASTGSKSGSSSSSGSRAGSSASSGSKSSSSASSGSKSSSSASTGSKSCSSASSGSKSSSSASTGSKSGSSSSSGSRAGSSASSG
SKSGSSASSASKSGISSSSGSRAGSSASSVSKSSGSRAGSSASSVSKSGSSASSGSKSGSSASSGSKSGSYSSSGSRAGSSASSVSKSS
GSRAGSSASSVSKSGSSASSGSKSGSSASSGSKSGSSSSSGSRAGSSASSVSKSGSSASSVSKSGVSSSSGSRVGSSASSGSKSGSSSS
SGSRAGSSASSGSKSGSSASSGSKSGSSASTGSKSGSSSSSGSRAGSSASSGSKSSSSASSGSKSSSSASTGSKSGSSSSSGSRAGSSA
SSGSKSGSSASSGSKSGSSSSSGSRAGSSASIGSKSGVSSSSGSRVGSSASSGSKSGSSASSGSKSCSSASSGSKSSSSASTGSKSGSS
SSSGSRAGSSASSGSKSSSSASSGSKSSSSASTGSKSGSSSSSGSRAGSSASSGSKSGSSASSASKSGISSSSGSRAGSSASSVSKSSG
SRAGSSASSVSKSGSSASSGSKSGSSASSGSKSGSSASSGSKSGSSSSSGSRAGSSASSGSKSGSSASSASKSGISSSSGSRAGSSASS
VSKSSGSRAGSSASSVSKLGSSASSGSKSGSSASSGSKSGSSSSSGSRAGSSASSVSKSSGSRAGSSASSVSKSGSSASSGSKSGSSAS
SGSKSGSSSSSGSRAGSSASSVSKSGSSASSVSKSGVSSSSGSRVGSSASSGSKSGSSSSSGSRAGSSASSVSKSGSSASSGSKSGSSA
SSGSKSGSSASSGSKSGSSASSGSKSGSSSSSGSRAGSSASSVSKSGSSASSVSKSGVSSSSGSRVGSSASSGSKSGSSSSSGSRAGSS
ASSVSKSGSSASSGSKSGSSASTGSKSGSSSSSGSRAGSSASSGSKSSSSASSGSKSSSSASTGSKSGSSSSGSRAGSSASSGSKSGS
SASSASKSGSSASSGSKSCSSASSGSKSSSSASTGSKSGSSSSSGSRAGSSASSGLKSGSSASSGSKSGSSASSGSKSGSSSSSGSRAG
SSASSVSKSSGSRAGSSASSVSKSSGSRAGSSASSVSKSGSSASSGSKSGSSASSGSKSGSSSSSGSRAGSSASSVSKSSGSRAGSSAS
SVSKSGSSASSGSKSGSSASSGSKSGSSSSSGSRAGSSASSVSKSGSSASSVSKSGVSSSSGSRVGSSASSGSKSGSSSSSGSRAGSSA
SSVSKSSGSRAGSSASSVSKSGSSASSGSKSCSSASSGSKSGSSASSGSKSSSSASTGSKSGSSSSSGSRAGSSASSGSKSSSSASSGS
KSSSSASTGSKSGSSSSSGSRAGSSASSGSKSGSSASSASKSGISSSSGSRAGSSASSVSKSSGSRAGSSASSVSKSGSSASSGSKSGS
SASSGSKSGSYSSSGSRAGSSASSVSKSSGSRAGSSASSVSKSGSSASSGSKSGSSASSGSKSGSSSSSGSRAGSSASSVSKSGSSSSS
GSRAGSSASSVSMSGSSASSGSKSGSSSSSGSRAGSSASSGSKSGSSASSGSKSRSSASSGSKSSSSASTGSKSGSSSSSGSRAGSSAS
SGSKSSSSASSGSKSSSSASSGSKSSSSASTGSKSGSSSSSGSRAGSSASSGSKSSSSASSGSKSSSSASTGSKSGSSSSGSRAGSSA
SSVSKSSGSRAGSSASSVSKSGSSASSGSKSGSSASSGSKSCSSASSGSKSSSSASTGSKSGSSSSSGSRAGSKSSSSASTGSKSGSSS
SSRSRAGSSASSGSKSGSSASSASKSGISSSSGSRAGSSASIGSKSGVSSSSGSRVGSSASSGSKSGSSAFSGSKSCSSASSVSKSSSS
ASTGSKSGSSSSSGSRAGSSASSGSKSSSSASSGSKSSSSASTGSKSGSSSSGSRVGSSASSGSKSGSSASSASKSCISSSSGSRAGS
SASSVSKSSGSRAGSSASSVSKSGSSASSGSKSGSSASSGSKSGSSSSSGSRAGSSASSVSKSSGSRAGSSASSVSKSGSSASSGSKSG
SSASSGSKSGSSSSSGSRAGSSASSVSKSGSSASSVSKSGVSSSSGSRVGSSASSGSKSGSSSSSGSRAGSSASSVSKSSGSRAGSSAS
SVSKSGSSASSGSKSCSSASSGSKSGSSASSGSKSCSSASSGSKSSSSASTGSKSGSSSSSGSRAGSSASSGSKSGSSASSGSKSCSSA
SSGSKSSSSASTGSKSGSYSSSGSRAGSSASSGSKSSSSSSSGSRAGSSASSGSKSGSSASSASKSGISSSSGSRAGSSASIGSKSGVS
SSSGSRAGSSASSVSKSSGSRAGSSASSVSKSGSSASSGSKSCSSASSGSKSGSSASSGSKSCSSASSGSKSSSASTGSKSGSSSSSG
SRAGSSASSGSKSSSSASSGSKSSSSASTGSKSGSSSSSGSRAGSSASSGSKSGSSASSASKSSISSSSGSRAGSSASSVSKSSGSRAG
SSASSVSKSGSSASSGSKSGSSASSGSKSGSSSSSGSRAGSSASSVSKSSGSRAGSSASSVSKSGSSASSGSKSGSSASSVSKSGSSAS
SGSKSGSSASSGSKSGISSSSGSRVGSSASSGSKSGSSASSGSKSSSSASTGSKSGSSSSSGSRAGSSASSGSKSGSSSSSGSRAGSSA
SSGSKSGSSASSGSKSGSSASSGSKSGSSSSSGSRAGSFASSGSKSGSSSSSGSRAGSSASSGSKSGSFSSSGSRAGSSASSGSKSGSS
SSSGSRAGSSASSGSKSGSSSSSGSRAGSSASSGSKSGSSSSIGSRAGSSSSSGSRAGSSASSGSKSGSSSSSGSRAGSSASSGSKSGS
SSSIGSRAGSSSSSGSRAGSSASSGSKLGCSSGRKSGRKYLSGRTLTSKKSYSNGKKSLVQRASKSRNKTDRKCEIGSLTTSKSKVKSN
IAGRTDSDSCQCHQKPRRNALKQIKNTLSAVSHNLAGLAEQIGTARSSVRSSKVSPKCNNVNGCHSQSSSTSSWTDEKGNVHRTAKSSG
SGAGRDSNGTPWSYNFDDGDNVNKDKISSGE

C) Sericin-like3(PV091730)

MRALSPTGLLATGVCLAIALNCDSSYASPFADRPQNLKIVEENKSETRDAKCDRSDVIVESSEKNKWNKKTNANKLCKKVEKREKQVSL
CHQLELIINKLKPKPLCHCKRIENSNTKYLGSKYESENKSTAKIKAASNAYNKKKQSISNSKNCNETKTIAKSCKKNKSTSKTKKAYSS
SYHTKAATSSWGKKYNCCSSDNINTSKEYNDLNTHKSTSSVSNNSNYSETNNSNESLNLKTRKQTSNQCNDLNTHKSTSSVSNNSNHSE
RNNSNESLNLKTRKQTSNQCNDLNTHKSTSSVSNNSNHSERNNSNESLNLKTRKQTSNQCNDLNTHKSTSSVSNNSKVCKKNTSNESLN
LKTRKQTSNQCNDLNTHKSTSSVSNNSNHSERNNSNESLNLKTRKQTSNQCNDLNTHKSTSSVSNNSNHSERNNSNESLNLKTRKQTSN
QCNDLNTHKSTSSVSNNSNVCKKNTSNESLNLKTRKQTSNQCNDLNTHKSTSSVSNNSNHSERNNSNESLNLKTRKQTSNQCNDLNSHK
STSSVSNNSNVCKKNTSNESLNLKTRKQTSNQCNDLNTHKSTSSVSNNSNHSERNNSNESLNLKTRKQTSNQCNDLNTHKSTSSVSNNS
NHSERNNSNESLNLKTRKQTSNQCNDLNTHKSTSSVSNNSKVCKKNTSNESLNLKTRKQTSNQCNDLNTHKSTSSVSNNSNHSERNNSN
ESLNLKTRKQTSNQCNDLNTHKSTSSVSNNSNHSERNNSNESLNLKTRKQTSNQCNDLNTHKSTSSVSNNSNVCKKNTSNESLNLKTRK
QTSNQCNDLNTHKSTSSVSNNSNHSERNNSNESLNLKTRKQTSNQCNDLNSHKSTSSVSNNSNVCKKNTSNESLNLKTRKQTSNQCNDL
NTHKSTSSVSNNSNHSERNNSNESLNLKTRKQTSNQCNDLNTHKSTSSVSNNSNHSETNNSNESLNLKTRKQTSNQCNDLNTHKSTSSV
SNNSNHSERNNANESLNLKTRKQTSNQCNDLNTHKSTSSVSNNSNVCKKNTSNESLNLKTRKQTSNQCNDLNTHKSTSSVSNNSNHSER
NNSNESLNLKTRKQTSNQCNDLNTHKSTSSVSNNSNVCKKNTSNESLNLKTRKQTSNQCNDLNTHKSTSSVSNNSKVCKKNTSNESLNL
KTRKQTSNQCNDLNTQKSTSSVSNNSNHSERNNSNESLNLKTRKQTSNQCNDLNTHKSTSSVSNNSNVCKKNTSNESLNLKTRKQTSNQ
CNDLNTHKSTSSVSNNSNVCKKNTSNESLNLKTRKQTSNQCNDLNTHKSTSSVSNNSNHSERNNSNESLNLKTRKQTSNQCNDLNTHKS
TSSVSNNSNVCKKNTSNESLNLKTRKQTSNQCNDLNTHKSTSSVYNNSNHSERNNSNESLNLKTRKQTSNQCNDLNTHKSTSSVSNNSN
VCKKNTSNESLNLKTRKQTSNQCNDLNTHKSTSSVSNNSNVCKKNTSNESLNLKTRKQTSNQCNDLNTHKSTSSVSNNSNVCKKNTSNE
SLNLKTRKQTSNQCNDLNTHKSTSSVSNNSNHSERNNSNESLNLKTRKQTSNQCNDLNTHKSTSSVSNNSNHSERNNSNESLNLKTRKQ
TSNQCNDLNTHKSTSSVSNNSNVCKKNNSNESLNLKTRKQTSNQCNDLNTHKSTSSVSNNSNVCKKNTSNESLNLKTRKQTSNQCNDLN
THKSTSSVSNNSNVCKKNTSNESLNLKTRKQTSNQCNDLNTHKSTSSVSNNSNHSERNNSNESLNLKTRKQTSNQCNDLNTHKSTSSVS
NNSNVCKKNTSNESLNLKTRKQTSNQCNDLNTHKSTSSVSNNSNHSERNNSNESLNLKTRKQTSNQCNDLNTHKSTSSVSNNSNHSERN
NSNESLNLKTCKQTSNQCNDLNTHKSTSSVSNNSNHSERNNSNESLNLKTRKQTSNQCNDLNTHKSTSSVSNNSNHSERNNSNESLNLK
TRKQTSNQCNDLNTHKSTSSVSNNSNVCKKNTSNESLNLKTRKQTSNQCNDLNTHKSTSSVSNNSNHSERNNSNESLNLKTRKQTSNQC
NDLNTHKSTSSVSNNSNVCKKNTSNESLNLKTRKQTSNQCNDLNTHKSTSSVSNNSNVCKKNTSNESLNLKTRKQTSNQCNDLNTHKST
SSVSNNSNVCKKNTSNESLNLKTRKQTSNQCNDLNTHKSTSSVSNNSKVCKKNTSNESLNLKTRKQTLNQCNDLNTHKSTSSVSNNSNH
SEKNNSNESLNLKTRKQTSNQCNDLNTHKSTSSVSNNSNHSERNNSNESLNLKTRKQTSNQCNDLNTHKSTSSVSNNSNVCKKNTSNES
LNLKTRKQTSNQCNDLNTHKSTSSVSNNSNVCKKNTTNESLNLKTRKQTSNQCNDLNTHKSTSSVSNNSNHSERNNSNESLNLKTRKQT
SNQCNDLNTHKSTSSVSNNSNVCKKNTSNESLNLKTRKQTSNQCNDLNTHKSTSSVSNNSNHSERNNSNESLNLKTRKQTSNQCNDLNT
HKSTSSVSNNSNHSERNNSNESLNLKTRKQTSNQCNDLNTHKSTSSVSNNSNVCKKNTSNESLNLKTRKQTSNQCNDLNTHKSTSSVSN
NSKVCKKNTSNESLNLKTRKQTSNQCNDLNTQKSTSSVSNNSNHSERNNSNESLNLKTRKQTSNQCNDLNTHKSTSSVSNNSNVCKKNT
SNESLNLKTRKQTSNQCNDLNTHKSTSSVSNNSNVCKKNTSNESLNLKTRKQTSNQCNDLNTHKSTSSVSNNSNHSERNNSNESLNLKT
RKQTSNQCNDLNTHKSTSSVYNNSNHSERNNSNESLNLKTRKQTSNQCNDLNTHKSTSSVSNNSNVCKKNTSNESLNLKTRKQTSNQCN
DLNTHKSTSSVSNNSNVCKKNTSNESLNLKTRKQTSNQCNDLNTHKSTSSVSNNSNVCKKNTSNESLNLKTRKQTSNQCNDLNTHKSTS
SVSNNSNHSERNNSNESLNLKTRKQTSNQCNDLNTHKSTSSVSNNSNHSERNNSNESLNLKTRKQTSNQCNDLNTHKSTSSVSNNSNVC
KKNNSNESLNLKTRKQTSNQCNDLNTHKSTSSVSNNSNHSERNNSNESLNLKTRKQTSNQCNDLNTHKSTSSVSNNSNHSERNNSNESL
NLKTRKQTSNQCNDLNTHKSTSSVSNNSNHSERNNSNESLNLKTRKQTSNQCNDLNAHKSTSSVSNNSNHSERNNSNESLNLKTRKQTS
NQCNDLNTHKSTSSVSNNSNHSERNNSNESLNLKTRKQTSNQCNDLNTHKSTSSVSNNSNVCKKNTSNESLNLKTRKQTSNQCNDLNTH
KSTSSVSNNSNHSERNNSNESLNLKTRKQTSNQCNDLNTHKSTSSVSNNSNVCKKNTSNESLNLKTRKQTSNQCNDLNTHKSTSSVSNN
SNHSERNNSNESLNLKTRKQTSNQCNDLNTHKSTSSVSNNSNHSERNNSNESLNLKTRKQTSNQCNDLNTHKSTSSVSNNSNHSERNNS
NESLNLKTRKQTSNQCNDLNTHKSTSSVSNNSNHSERNNSNESLNLKTRKQTSNQCNDLNTHKSTSSVSNNSNHSERNNSNESLNLKTR
KQTSNQCNDLNTHKSTSSVSNNSNVCKKNTSNESLNLKTRKQTSNQCNDLNTHKSTSSVSNNSKVCKKNTSNESLNLKTRKQTSNQCND
LNTQKSTSSVSNNSNHSERNNSNESLNLKTRKQTSNQCNDLNTHKSTSSVSNNSNVCKKNTSNESLNLKTRKQTSNQCNDLNTHKSTSS
VSNNSNVCKKNTSNESLNLKTRKQTSNQCNDLNTHKSTSSVSNNSNHSERNNSNESLNLKTRKQTSNQCNDLNTHKSTSSVSNNSNVCK
KNTSNESLNLKTRKQTSNQCNDLNTHKSTSSVSNNSNHSERNNSNESLNLKTRKQTSNQCNDLNTHKSTSSVSNNSNVCKKNTSNESLN
LKTRKQTSNQCNDLNTHKSTSSVSNNSNVCKKNTSNESLNLKTRKQTSNQCNDLNTHKSTSSVSNNSNHSERKTSNESLNLKTRKQTSN
QCNDLNTHKSTSSVSNNSNHSERNNSNESLNLKTRKQTSNQCNDLNTHKSTSSVSNNSNHSERNNSNESLNLKTRKQTSNQCNDLNTHK
STSSVSNNSNHSERNNSNESLNLKTRKQTSNQCNDLNAHKSTSSVSNNSNHSERNNSNESLNLKTRKQTSNQCNDLNTHKSTSSVSNNS
NVCKKNTSNESLNLKTRKQTSNQCNDLNTHKSTSSVSNNSNHSERNNSNESLNLKTRKQTSNQCNDLNTHKSTSSVSNNSNVCKKNTSN
ESLNLKTRKQTSNQCNDLNTHKSTSSVSNNSNHSERNNSNESLNLKTRKQTSNQCNDLNTHKSTSSVSNNSNVCKKNTSNESLNLKTRK
QTSNQCNDLNTHKSTSSVSNNSNHSERNNSNESLNLKTRKQTSNQCNDLNTNKSTSSVSNNSNHSERNNSNESLNLKTRKQTSNQCNDL
NTHKSTSSLSNNSNHSERNTSNESLNLKTRKQTSNQCNDLNTHKSTSSVSNNSNVCKKNTSNESLNLKTRKQTSNQCNDLNTHKSTSSV
SNNSNVCKNNTSNESLNLKTRKQTSNQCNDLNTHKSTSSVSNNSNHSERNNSNESLNLKTRKQTSNQCNDLNTHKSTSSVSNNSNHSER
NNSNESLNLKTRKQTSNQCNDLNTHKSTSSVSNNSNHSERNNSNESLNLKTRKQTSNQCNDLNTHKSTSSVSNNSNHSERNNSNESLNL
KTRKQTSNQCNDLNTHKSTSSVSNNSNVCKKNTSNESLNLKTRKQTSNQCNDLNTHKSTSSVSNNSNVCKKNTSNESLNLKTRKQTSNQ
CNDLNTHKSTSSVSNNSNVCKKNTSNESLNLKTRKQTSNQCNDLNTHKSTSSVSNNSNHSERNNSNESLNLKTRKQTSNQCNDLNTHKS
TSSVSNNSNVCKKNTSNESLNLKTRKQTSNQCNDLNTHKSTSSVSNNSNHSERNNSNESLNLKTRKQTSNQCNDLNTHKSTSSVSNNSN
VCKKNTSNESLNLKTRKQTSNQCNDLNTHKSTSSVSNNSNVCKKNTSNESLNLKTRKQTSNQCNDLNTHKSTSSVSNNSNHSERKTSNE
SLNLKTRKQTSNQCNDLNTHKSTSSVSNNSNHSERNNSNESLNLKTRKQTSNQCNDLNTHKSTSSVSNNSNHSERNNSNESLNLKTRKQ
TSNQCNDLNTHKSTSSVSNNSNHSERNNSNESLNLKTRKQTSNQCNDLNAHKSTSSVSNNSNHSERNNSNESLNLKTRKQTSNQCNDLN
THKSTSSVSNNSNVCKKNTSNESLNLKTRKQTSNQCNDLNTHKSTSSVSNNSNHSERNNSNESLNLKTRKQTSNQCNDLNTHKSTSSVS
NNSNVCKKNTSNESLNLKTRKQTSNQCNDLNTHKSTSSVSNNSNHSERNNSNESLNLKTRKQTSNQCNDLNTHKSTSSVSNNSNVCKKN
TSNESLNLKTRKQTSNQCNDLNTHKSTSSVSNNSNHSERNNSNESLNLKTRKQTSNQCNDLNTNKSTSSVSNNSNHSERNNSNESLNLK
TRKQTSNQCNDLNTHKSTSSLSNNSNHSERNTSNESLNLKTRKQTSNQCNDLNTHKSTSSVSNNSNVCKKNTSNESLNLKTRKQTSNQC
NDLNTHKSTSSVSNNSNVCKNNTSNESLNLKTRKQTSNQCNDLNTHKSTSSVSNNSNHSERNNSNESLNLKTRKQTSNQCNDLNTHKST
SSVSNNSNHSERNNSNESLNLKTRKQTSNQCNDLNTHKSTSSVSNNSNHSERNNSNESLNLKTRKQTSNQCNDLNTHKSTSSVSNNSNH
SERNNSNESLNLKTRKQTSNQCNDLNTHKSTSSVSNNSNVCKKNTSNESLNLKTRKQTSNQCNDLNTHKSTSSVSNNSNVCKKNTSNES
LNLKTRKQTSNQCNDLNTHKSTSSVSNNSNHSERNNSNESLNLKTRKQTSNQCNDLNTHKSTSSVSNNSNVCKKNTSNESLNLKTRKQT
SNQCNDLNTHKSTSSVSNNSNVCKKNTSNESLNLKTRKQTSNQCNDLNTHKSTSSVSNNSNHSERNNSNESLNLKTRKQTSNQCNDLNT

HKSTSSVSNNSNVCKKNTSNESLNLKTRKQTSNQCNDLNTHKSTSSVSNNSNVCKKNTSNESLNLKTRKQTSNQCNDLNTHKSTSSVSN
NSNHSERNNSNESLNLKTRKQTSNQCNDLNTHKSTSSVSNNSNHSERNNSNESLNLKTRKQTSNQCNDLNTHKSTSSVSNNSNHSERNN
SNESLNLKTRKQTSNQCNDLNTHKSTSSVSNNSNHSERNNSNESLNLKTRKQTSNQCNDLNTHKSTSSVSNNSNHSERNNSNESLNLKT
RKQTSNQCNDLNTHKSTSSVSNNSNHSERNNSNESLNLKTRKQTSNQCNDLNTHKSTSSVSNNSNVCKKNNSNESLNLKTRKQTSNQCN
DLNTHKSTSSVSNNSNHSERNNSNESLNLKTRKQTSNQCNDLNTHKSTSSVSNNSNVCKKNTSNESLNLKTRKQTSNQCNDLNTHKSTS
SVSNNSNHSERNNSNESLNLKTRKQTSNQCNDLNTHKSTSSVSNNSNHSERNNSNESLNLKTRKQTSNQCNDLNTHKSTSSVSNNSNHS
ERNNSNESLNLKTRKQTSNQSNDLKTNKSTSSVSNNSNHSERNNSNESLNLKTRKQTSNQCNDLNTHKSTSSVSNNSNHSERNNSNESL
NLKTRKQTSNQCNDLNTHKSTSSVSNNSNVCKKNTSNESLNLKTRKQTSNQCNDLNTHKSTSSVSNNSNVCKKNTSNESLNLKTRKQTS
NQCNDLNTHKSTSSVSNNSNHSERNNSNESLNLKTRKQTSNQCNDLNTHKSTSSVSNNSNVCKKNTSNESLNLKTRKQTSNQCNDLNTH
KSTSSVSNNSNVCKKNTSNESLNLKTRKQTSNQCNDLNTHKSTSSVSNNSNHSERNNSNESLNLKTRKQTSNQCNDLNTHKSTSSVSNN
SNHSERNNSNESLNLKTRKQTSNQCNDLNTHKSTSSVSNNSNHSERNNSNESLNLKTRKQTSNQCNDLNTHKSTSSVSNNSNHSERNNS
NESLNLKTRKQTSNQCNDLNTHKSTSSVSNNSNHSERNNSNESLNLKTRKQTSNQCNDLNTHKSTSSVSNNSNHSERNNSNESLNLKTR
KQTSNQCNDLNTNKSTSSVSNNSNHSERNNSNESLNLKTRKQTSNQCNDLNTHKSTSSVSNNSNVCKKNTSNESLNLKTRKQTSNQCND
LNTHKSTSSVSNNSNHSERNNSNESLNLKTRKQTSNQCNDLNTHKSTSSVSNNSNVCKNNTSNESLNLKTRKQTSNQCNDLNTHKSTSS
VSNNSNHSERNNSNESLNLKTRKQTSNQCNDLNTHKSTSSVSNNSNHSERNNSNESLNLKTRKQTSNQCNDLNTHKSTSSVSNNSNHSE
RNNSNESLNLKTRKQTSNQCNDLNTHKSTSSVSNNSNHSERNNSNESLNLKTRKQTSNQCNDLNTNKSTSSVSNNSNHSERNNSNESLN
LKTRKQTSNQCNDLNTHKSTSSVSNNSNHSERNNSNESLNLKTRKQTSNQCNDLNTHKSTSSVSNNSNVCKKNTSNESLNLKTRKQTSN
QCNDLNTHKSTSSVSNNSNHSERNNSNESLNLKTRKQTSNQCNDLNTHKSTSSVSNNSNHSERNNSNESLNLKTRKQTSNQCNDLNTHK
STSSVSNNSNHSERNNSNESLNLKTRKQTSNQCNDLNTHKSTSSVSNNSNHSERNNSNESLNLKTRKQTSNQCNDLNTHKSTSSVSNNS
NHSERNNSNESLNLKTRKQTSNQCNDLNAHKSTSSVSNNSNHSERNNSNESLNLKTRKQTSNQCNDLNTHKSTSSVSNNSNVCKKNTSN
ESLNLKTRKQTSNQCNDLNTHKSTSSVSNNSNVCKKNTSNESLNLKTRKQTSNQCNDLNTHKSTSSVSNNSNHSERNNSNESLNLKTRK
QTSNQCNDLNTHKSTSSVSNNSKVCKKNTSNESLNLKTRKQTSNQCNDLNTHKSTSSVSNNSNHSERNNSNESLNLKTRKQTSNQCNDL
NTHKSTSSVSNNSNVCKKNTSNESLNLKTRKQTSNQCNDLNTHKSTSSVSNNSNHSERNNSNESLNLKTRKQTSNQCNDLNTHKSTSSV
SNNSNVCKKNTSNESLNLKTRKQTSNQCNDLNTHKSTSSVSNNSNHSERNNSNESLNLKTRKQTSNQCNDLNTHKSTSSVSNNSNVCKK
NTSNESLNLKTRKQTSNQCNDLNTHKSTSSVSNNSNVCKNNTSNESLNLKTRKQTSNQCNDLNTHKSTSSVSNNSNHSERNNSNESLNL
KTRKQTSNQCNDLNTHKSTSSVSNNSNVCKKNTSNESLNLKTRKQTSNQCNDLNTHKSTSSVSNNSNVCKKNTSNESLNLKTRKQTSNQ
CNDLNTHKSTSRVSNNSNVCKKNTSNESLNLKTRKQTSNQCNDLNTHKSTSSVSNNSNVCKKNTSNKSLNLKTRKQTSNQCNDLNTHKS
TSSVSNNSNHSERNNSNESLNLKTRKQTSNQCNDLNTHKSTSSVSNNSNHSERNNSNESLNLKTRKQTSNQCNDLNTHKSTSSVSNNSN
HSERNNSNESLNLKTRKQTSNQCNDLNTHKSTSSVSNNSNVCKNNTSNESLNLKTRKQTSNQCNDLNTHKSTSSVSNNSNICKKNTSNE
SLNLKTRKQTSNQCNDLNTHKSTSSVSNNSNVCKKNTSNESLNLKTRKQTSNQCNDLNTHKSTSSVSNNSNVCKKNTSNESLNLKTRKQ
TSNQCNDLNTHKSTSSVSNNSNHSERNNSNESLNLKTRKQTSNQCNDLNTHKSTSSVSNNSNHSERNNSNESLNLKTRKQTSNQCNDLN
THKSTSNVSNNSNHSERNNSNESLNLKTRKQTSNQCNDLNTHKSTSSVSNNSNVCKKNTSNESLNLKTRKQTSNQCNDLNTHKSTSSVS
NNSNFCKKNTSNESLNLKTRKQTSNQCNDLNTHKSTSSVSNNSNVCKKNTSNESLNLKTRKQTSNQCNDLNTHKSTSSVSNNSKVCKKN
TSNESLNLKTRKQTSNQCNDLNTHKSTSSVSNNSNHSERNNSNESLNLKTRKQTSNQCNDLNTNKSTSSVSNNSNHSERNNSNESLNLK
TRKQTSNQCNDLNTHKSTSSVSNNSNHSERNTSNESLNLKTRKQTSNQCNDLNTHKSTSSVSNNSNVCKKNTSNESLNLKTRKQTSNQC
NDLNTHKSTSSVSNNSNVCKNNTSNESLNLKTRKQTSNQCNDLNTHKSTSSVSNNSNHSERNNSNESLNLKTRKQTSNQCNDLNTHRST
SSVSNNSNVCKKNTSNESLNLKTRKQTSNQCNDLNTHKSTSSVSNNSNVCKKNTSNESLNLKTRKQTSNQCNDLNTHKSTSSVSNNSNV
CKKNTSNESLNLKTRKQTSNQCNDLNTHRSTSSVSNNSNHSERNNSNESLNLKTRKQTSNQCNDLNTHKSTSSVSNNSNHFERNNSNES
LNLKTRKQTSNQCNDLNTHKSTSSVSNNSNHSERNNSNESLNLKTRKQTSNQCNDLNTHKSTSSVSNNSNVCKKNTSNESLNLKTRKQT
SNQCNDLNTHKSTSSVSNNSNHSERNNSNESLNLKTRKQTSNQCNDLNTHKSTSSVSNNSNVCKKNTSNESLNLKTRKQTSNQCNDLNT
HKSTSSVSNNSNHFERNNSNESLNLKTRKQTSNQCNDLNTHKSTSSVSNNSNVCKKNTSNESLNLKTRKQTSNQCNDLNTHKSTSSVSN
NSNHSERNNSNESLNLKTRKQTSNQCNDLNTHKSTSSVSNNSNHSERNNSNESLNLKTRKQTSNQCNDLNTHKSTSSVSNNSNVCKKNT
SNESLNLKTRKQTSNQCNDLNTHKSTSSVSNNSNHSERNNSNESVNLKTRKQTSNQCNDLNTHKSTSSVSNNSNVCKKNTSNESLNLKT
RKQTSNQCNDLNTHKSTSSVSNNSNHSERNNSNESLNLKTRKQTSNQCNDLNTHKSTSSVSNNSNHSERNNSNESLNLKTRKQTSNQCN
DLNTHKSTSSVSNNSNHSERNNSNESLNLKTRKQTSNQCNDLNTHKSTSSVSNNSNHSERNNSNESLNLQTRKQTSNQCNDLNTHKSTS
SVSNNSNHSERNNSNESLNLKTRKQTSNQCNDLNTHKSTSSVSNNSNVCKKNTSNESLNLKTRKQTSNQCNDLNTHKSTSSVSNNSNHS
ERNNSNESLNLKTRKQTSNQCNDLNTHKSTSSVSNNSNVCKKNTSNESLNLKTRKKTSNQCNDLNTHKSTSSVSNNSNVCKKNTSNESL
NLKTRKQTSNQCNDLNTHKSTSSVSNNSNFCKKNTSNESLNLKTRKQTSNQCNDLNTHKSTSSVSNNSNHSERNNSNESLNLQTRKQTS
NQCNDLNTHKSTSSVSNNSNHSERNNSNESLNLKTRKQTSNQCNDLNTHKSTSSVSNNSNVCKKNTSNESLNLKTRKQTSNQCNDLNTH
KSTSSVSNNSNVCKKNTSNESLNLKTRKQTSNQCNDLNTHKSTSSVSNNSNFCKKNTSNESLNLKTRKQTSNQCNDLNTHKSTSSVSNN
SNHSERNNSNESLNLKTRKQTSNQCNDLNTHKSTSSVSNNSNVCKKNTSNESLNLKTRKQTSNQCNDLNTHKSTSSVSNNSNHSERNNS
NESLNLKTRKQTSNQCNDLNTHKSTSSVSNNSNHSERNNSNESLNLKTRKQTSNQCNDLKTNKSTSSVSNNSNHSERNNSNESLNLKTR
KQTSNQCNDLNTHKSTSSVSNNSNHSERNNSNESLNLKTRKQTSNQCNDLNTHKSTSSVSNNSNVCKKNTSNESLNLKTRKQTSNQCND
LNTHKSTSSVSNNSNHSERNNSNESLNLKTRKQTSNQCNDLNTHKSTSSVSNNSNHSERNNSNESLNLKTRKQTSNQCNDLNTHKSTSS
VSNNSNHSERNNSNESLNLKTRKQTSNQCNDLNTHKSTSSVSNNSNHSERNNSNESLNLKTRKQTSNQCNDLNTHKSTSSVSNNSNHSE
RNNSNESLNLKTRKQTSNQCNDLNTHKSTSSVSNNSNHSERNNSNESLNLKTRKQTSNQCNDLNTHKSTSSVSNNSNVCKKNTSNESLN
LKTRKQTSNQCNDLNTHKSTSSVSNNSNHSERNNSNESLNLQTRKQTSNQCNDLNTHKSTSSVSNNSNHSERNNSNESLNLKTRKQTSN
QCNDLNTHKSTSSVSNNSNHSERNNSNESLNLKTRKQMSNQCNDLNTHKSTSSVSNNSNHSERNNSNESLNLKTRKQTSNQCNDLNTHK
STSSVSNNSNHSERNNSNESLNLKTRKQTSNQCNDLNTHKSTSSVSNNSNHSERNNSNESLNLKTRKQTSNQCNDLNTHKSTSSVSNNS
NHSERNNSNESLNLKTRKQTSNQCNDLNTHKSTSSVSNNSNHSERNNSNESLNLKTRKQTSNQCNDLNTHKSTSSVSNNSNHSERNNSN
ESLNLKTRKQTSNQCNDLNTHKSTSSVSNNSNHSERNNSNESLNLKTRKQTSNQCNDLNTHKSTSSVSNNSNHSERNNSNESLNLKTRK
QTSNQCNDLNTHKSTSSVSNNSKVCKKNTSNESLNLKTRKQTSNQCNDLNTHKSTSSVSNNSNHSERNNSNESLNLKTRKQTSNQCNDL
NTHKSTSSVSNNSNHSERNNSNESLNLKTRKQTSNQCNDLNTHRSTSSVSNNSNVCKKNTSNESLNLKTRKQTSSQCNDLNTHKSTSSV
SNNSNHSERNNSNESLNLKTRKQTSNQCNDLNTHKSTSSVSNNSNHSERNNSNESLNLKTRKQTSNQCNDLNTHKSTSSVSNNSNHSER
NNSNESLNLKTRKQTSNQCNDLNTHKSTSSVSNNSNVCKKNTSNESLNLKTRKQTSNQCNDLNTHKSTSSVSNNSNVCKKNTSNESLNL
KTRKQTSNQCNDLNTHKSTSSVSNNSNHSERNNSNESLNLKTRNQTSNQCNDLNTNKSTSSVSNNSNHSERNNSNESLNLKTRKQTSNQ
CNDLNTHKSTSSVSNNSNHSERNNSNESLNLKTRKQTSNQCNDLNTHKSTSSVSNNSNVCKKNTSNESLNLKTRKQTSNQCNDLNTHKS
TSSVSNNSNHSERNNSNESLNLKTRKQTSNQCNDLNTHKSTSSVSNNSNVCKKNTSNESLNLKTRKQTSSQCNDLNTHKSTSSVSNNSN
HSERNNSNESLNLKTRKQTSNQCNDLNTHKSTSSVSNNSNHSERNNSNESLNLKTRKQTSNQCNDLNTHKSTSSVSNNSNHSERNNSNE
SLNLKTRKQTSNQCNDLNTNKSTSSVSNNSNHSERNNSNESLNLKTRKQTSNQCNDLNTHKSTSSVSNNSNHSERNNSNESLNLKTRKQ
TSNQCNDLNTHKSTSSVSNNSNVCKKNTSNESLNLKTRKQTSNQCNDLNTHKSTSSVSNNSNHSERNNSNESLNLKTRKQTSNQCNDLN

THKSTSSVSNNSNHSERNNSNESLNLKTRKQTSNQCNDLNTHKSTSSVSNNSNHSERNNSNESLNLKTRKQTSNQCNDLNTHKSTSSVS
NNSNVCKKNTSNESLNLKTRKQTSNQCNDLNTHKSTSSVSNNSNHSERNNSNESLNLKTRKQTSNQCNDLNTHKSTSSVSNNSNVCKKN
TSNESLNLKTRKQTSNQCNDLNTHKSTSSVSNNSNHSERNNSNESLNLKTRKQTSNQCNDLNTHKSTSSVSNNSNHSERNNSNESLNLK
TRKQTSNQCNDLNTHKSTSSVSNNSNVCKKNTSNESLNLKTRKQTSNQCNDLNTHKSTSSVSNNSNHSERNNSNESLNLKTRKQTSNQC
NDLNTHKSTSSVSNNSNVCKNNTSNESLNLKTRKQTSNQCNDLNTHKSTSSVSNNSNICKKNTSNESLNLKTRKQTSNQCNDLNTHKST
SSVSNNSNICKKNTSNESLNLKTRKQTSNQCNDLNTHKSTSSVSNNSNHSERNNSNESLNLKTRKQTSNQCNDLNTYKSASCESIDSNF
GAISGDKHDLSVSDNSNTHINTLNPYSRCKSLCFVNALHSSARSLCNNNLGSTSKALCKKRPRRRNLRRNLSSRRKSIITKSVSVSNSK
RKSMACSEKRNKTLACSKSIGRCSSRKSHNNSESHSHSKSNTTPKSPRCLRSGPATQNDSDLRQCHSTTSFNAFKHIDGVLSGVSEDLV
KHAGCAPRSMSHLHSSVNDNNCRQTDVGPSISKSSDCGSSSWNHNINSGDNANADKISSGG

D) Cadhezin-like 1 (PV091731)

MRILAAVLGCTLLLLAFLSWAESTPSISLNPISEDFNSAQTRQRRNAFDFIVDSAANIAKSVADVAVTLVGTALALIAGFLKAIGVVVF
GFASALLSLPGGLPEGVVLPDITALQNGEIKDPNEAILIITALIKASNMIIVKGNSDQKDYLLDKMTIYLPKITDELKKILEPSDLEEL
CKVLSLKSFRDLLVEYNDMKINLQTLKSTSKTLLLISLVEETGEIITKIISYIEIYTISFKNDNSEVDDTTAHVDTTESAFDEVLDGVI
EESVTSTHTPSLSETDSEESVAIELDLNTNEIDDEHIADKDLHLDFETEDVYVNIDEQKEISGHESAVISIDDYVLLLTKFLSLVSGNE
KEISAVLAGAIVSTEHGYHASLAVLLDNISEISALFEGVNKEKLLSLQVSLKQLQVEVLKLKLSDVVTALLQKITVAVNVVTSVLKLVE
NLNVAALELSLNAFVIAIETGTPAQREAILRGENIDAPGGLKVSLIFILENISKIHLNTENITRERLVALMTLLKQLRVLLTKYESAHV
ETLSKKIYESLITLDMVLRTREQLELNNLTKFLFNIVFIIKSGDAPAIKNIITGGLLPELGGGTIKLSLLDALNRLAVILGTNQDLPLS
KILRLKYASLDLKVALLDLKTTEFAQSRILLEKLLLIQDLVKKPIELSEIFNPDITTEFLKIFNKILTSGNIAQINEVLSGKDFTGPNN
LKISLQSLLDIVFTFNLEMKYTSTTTLVIMQSVLSDLHDVLRERAQTVETVNIISKLSFALHLVSELIAHFDLLSVEFYKDYIIMILTA
LKGASPTRINEIITGDRFVGSHGISLSVFTLMDKLVDLKVTLGGISLDGLDQLKEAMLKLKTELNKLPKTNSKAVEILNTIDVALTVNI
NMHTEIKAINIVVINQFLSLMVNANAETIKTIISGGKITGANGKQLSLIEILNLISGLKHSSEGVNKAAIATLLKGLHGIYDLLYKLPK
THPLLNYLLRRLTITIYAVRTIAVYEARLGLEALTPVVTSFIRIINRAPVDRKDAIIARGVIKLAGGVQISFRHIVEYILLRRSSDRGL
TIRKLVNMREKFVRMRRLLWRIKRTWVVTRALRRIAKVLNHLDLLAREVRTLNLLQLLRAINNLLKNGTSNQKHAIVNGGVVSTPYLRG
SLQATLAAMRRQPSLLRFRWLQYRHLLTRQKNELIKSKNYLKNLSFAPAALIKNVVNTYGLLNAYFLQRA

E) Cadhezin-like 2 (PV091732)

MKLIVFICSAFALSLCLNFAESSPFHFGGIHLPGLPKIETHTPKDSFTNLFGKAINTASKITGGIVNGIAGLAGIGLKLLSGFLRSIGG
AAWSLANLLKGLPGGLPEGIVIPDLSGLNDVKDANKAIITLANLIEVINEIFKRGNSLQINALLSDDGLLVILPKLLTAIETLISEPGH
LNELNVAISIDSIRRLFSSLNSLKINIQANISVNGAKELIVIIDKALQLLNGVSTHLFPTLITDLVKGLRELALILVGSLQGIPGGLPS
GLVITDISLLTSNKITDANEAITIITKLFNIFNEVLINGNSLQINACLDGKTGLQAILPKLIAFLKSLLSDKEKLLKLNVVISIESMSK
LYSVIGQLGITADAKMPNTTVLIEMFNEAQLLINKMTSFLYPYLTSSLISNLKNVIQALLLSITNISTGLPVGIKIPEVNNLINGNIVD
ANEAFYILIDIFDVFNAILTQGNNQQINALLTEKDGIQFTLPKLVALLKTLISNQENFKELNVVVSYDTLKRLGIALGLFQFNIPHTAN
NDVTLILNKYIKESQNSLNKITAQLYPEIVAILIKKIGNLAISLSKSIYDLPGGLPSSIIIPDVSILSKQSDPNDAFDLVTKIIQTYNQ
VLELGDTSQIYALISNKDGLLKSLPNIIPFLESLLSGTTINLKTFSVIISGETLNNFYSALTRLRVNIETKASVESSKILISIIDKALQ
LLKEIKTITFPQLFSGLIKSFGNLALSLVDSFTDLPGGLPNGIIMPDVTIFNGSNLPKINVAFNLLIEIFETFMAVLNKGNPLQITAVL
TGKNGLQVKLTQIITFLNDLSYPANLKSLNIVITLGGLNKFYTTFALIKENIIKTDIINNSMVLTEAIDNILTLVNQITSTMFPDLNYY
YLQELRNLGTLLINSFNGLPEGLPTGITIPNGDAKLTNLDDAFVFVANLLETYPAAVKHLNPLQISDFLTGKGGLKAILPIIVDFFKDL
IRNPAHLISLKTAVSLESLNKLHSALTQLREVVSFSVKLTAQIDETLTLLEHFASHLFPVPVSVLIEGIANIGALLVNSFTGLPGGLPT
GITIPNGNAKLTNLDEAFVFVAKLLETYPAAVKHLSPSEISAFLSGKGGLEAVLPNIVAFLKDLLRNPAQLKSLKTAVSLESINKLHSA
LTQLREVVSFSVKLTAQIDETLTLLEHFASHLFPVPVSELIEGIANIGALLVNSFTGLPGGLPTGITIPNGNAKLTNLDEAFVFVAKLL
ETYPAAVKHLSPSEISAFLSGKGGLEAVLPNIVAFLKDLLRNPAQLKSLKTAVSLESINKLHSALTQLREVVSFSVKLTAQIDETLTLL
EHFASHLFPVPVSVLIEGIANIGALLVNSFTGLPGGLPTGITIPNGNAKLTNLDEAFVFVAKLLETYPVAVKHLSPSEISAFLSGKGGL
EAVLPNIVAFLKDLLRNPAQLKSLKTAVSLESLNKLHSALTQLREVVSFSVKLTAQIDETLTLLEHFASHLFHVPVSELIEGIANIGAL
LVNSFTGLPGGLPTGITIPNGNAKLTNLDEAFVFVAKLLETYPAAVKHLSPSEISAFLSGKGGLEAVLPNIVAFLKDLLRNPAQLKSLK
TAVSLESINKLHSALTQLREVVSFSVKLTAQIDETLTLLEHFASHLFPVPVSELIEGIANIGALLVNSFTGLPGGLPTGITIPNGNAKL
TNLDEAFVFVAKLLETYPAAVKHLSPSEISAFLSGKGGLEAVLPNIVAFLKDLLRNPAQLKSLKTAVSLESINKLHSALTQLREVVSFS
VKLTAQIDETLTLLEHFASHLFPVSVSELIEGIANIGALLVNSFTGLPEGLPTGITIPNGNAKLTNLDEAFVFVAKLLETYPVAVKHLS
PSEISAFLSGKGGLEAVLPNIVAFLKDLLRNPAQLKSLKTAVSLESLNKLHSALTQLREVVSFSVKLTAQIDETLTLLEHFASHLFPVP
VSVLIEGIANIGALLVNSFTGLPGGLPTGITIPNGNAKLTNLDEAFVFVAKLLETYPAAVKHLSPSEISAFLSGKGGLEAVLPNIVAFL
KDLLRNPAQLKSLKTAVSLESINKLHSALTQLREVVSFSVKLTAQIDETLTLLEHFASHLFPVPVSVLIEGIANIGALLVNSFTGLPGG
IPTGITLPNGNAKLTNLDEAFVFVAKLLETYPAAVKHLSPSEISAFLSGKGGLEAVLPNIVAFLKDLLKHPAHLKSLNIPLSPESINKL
ESALVLLRASIEVASPEIYRKFTTQIDEILTLFKQFASHVIPVSVSGLIEGIANIGALLVNSFTGLPGGLPTGITIPTGTVKLANLDEA
FVFVAKLLETYPAAVKDLSPVQISAFLSGKGGLEAVLPNIVAFLKDLLRNPAQLKSLKTAVSLESLNKLHSALTQLREVVSFSVKLTAQ
IDETLTLLEHFASHLFPVPVSVLIEGIANIGALLVNSFTGLPGGLPTGITIPNGNAKLTNLDEAFVFVAKLLETYPAAVKHLSPSEISA
FLSGKGGLEAVLPNIVAFLKDLLRNPAQLKSLKTAVSLESLNKLHSALTQLREVVSFSVKLTAQIDETLTLLEHFASHLFPVPVSVLIE
GIANIGALLVNSFTGLPGGIPTGITLPNGNAKLTNLDEAFVFVAKLLETYPAAVKHLSPSEISAFLSGKGGLEAVLPNIVAFLKDLLRN
PAQLKSLKTAVSLESLNKLHSALTQLREVVSFSVKLTAQIDETLTLLEHFASHLFPVPVSVLIEGIANIGALLVNSFTGLPGGLPTGIT
IPNGNAKLTNLDEAFVFVAKLLETYPAAVKHLSPSEISAFLSGKGGLEAVLPNIVAFLKDLLRNPAQLKSLKTAVSLESINKLHSALTQ
LREVVSFSVKLTAQIDETLTLLEHFASHLFPVPVSVLIEGIANIGALLVNSFTGLPGGIPTGITLPNGNAKLTNLDEAFVFVAKLLETY
PAAVKHLSPSEISAFLSGKGGLEAVLPNIVAFLKDLLKHPAHLKSLNIPLSPESINKLESALVLLRASIEVASPEIYRKFTTQIDEILT
LFKQFASHVIPVSVSGLIEGIGNIGVLLVNSFTSLPGGLPTGITIPTGTVKLANLDEAFVFVAKLLETYPAAVKDLSPVQISAFLSGKG
GLEAVLSNIVAFLTDLLKHPAHLKSLNIPLSPESINKLESALVLLRASIIEVASPEISRKFTTQIDEILTLLKQFASHVIPVSVSGLLE
GIGNIGISLVNSFTSLPGGLPTGITIPTGTVKLANLDEAFVFVAKLLETYPAAVKDLSPVQINAFLSGKGGLEAVLSNIVAFLTDLLKH
PAHLKSLNIPLSPESINKLESALVLLRASIIEVASPEISRKFTTQIDEILTLLKQFASHVIPVSVSGLLEGIGNIGISLVNSFTSLPGG
LPTGITIPTGTVKLANLDEAFVFVAKLLETYPAAVKDLSPVQISAFLSGKGGLEAVLSNIVAFLTDLLKHPAHLKSLNIPLSPESINKL
ESALVLLRASIIEVASPEISRKFTTQIDEILTLLKQFASHVIPVSVSGLLEGIGNIGISLVNSFTSLPGGLPTGITIPTGTVKLANLDE

AFVFVAKLLETYPAAVKDLSPVQISAFLSGKGGLEAVLSNIVAFLKDLLKHPAHLKSLNIPLSPESINKLESALVLLRASIIDVASPEI
SRKFTTQIDEILTLLKQFASHVIPVSVSGLLEGIGNIGISLVNSFTSLPGGLPTGITIPTGTVKLANLDEAFVFVAKLLETYPAAVKDL
SPVQISAFLSGKGGLEAVLSNIVAFLKDLLKHPAHLKSLNIPLSPESINKLESALVLLRASIIEVASPEISRKFTTHIDEILTLLKQFA
SHVIPVSVSGLLEGIGNIGISLVNSFTSLPGGLPTGITIPTGTVKLANLDEAFVFVAKLLETYPAAVKDLSPVQISAFLSGKGGLEAVL
SNIVAFLKDLLKHPAHLKSLNIPLSPESIKKLESALVLLRASIIDVASPEISRKFTTQIDEILTLLKQFASHVIPVSVSGLLEGIGNIG
ISLVNSFTSLPGGLPTGITIPTGTVKLANLDEAFVFVAKLLETYPAAVKDLSPVQISAFLSGKGGLEAVLSNIVAFLKDLLKHPAHLKS
LNIPLSPESINKLESALVLLRASIIDVASPEISRKFTTQIDEILTLLKQFASHVIPVSVSGLLEGIGNIGISLVNSFTSLPGGLPTGIT
IPTGTVKLANLDEAFVFVAKLLETYPAAVKHLSPSEISAFLSGKGGLEAVLSNIVAFLTDLLKHPAHLKSLNIPLSPESINKLESALVL
LRASIIEVASPEISRKFTTQIDEILTLLKQFASHLISVSVSGLLEGIGNIGISLVNSFTSLPGGLPTGITIPTGTVKLANLDEAFVFVA
KLLETYPAAVKDLSPVQISAFLSGKGGLEAVLSNIVAFLKDLLKHPAHLKSLNIPLSPESINKLESALVLLRASIIDVASPEISRKFTT
QIDEILTLLKQFASHVIPVSVSGLLEGIGNIGISLVNSFTSLPGGLPTGITIPTGTVKLANLDEAFVFVAKLLETYPAAVKDLSPVQIS
AFLSGKGGLEAVLSNIVAFLTDLLKHPAHLKSLNIPLSPESINKLESALVLLRASIIEVASPEISRKFTTQIDEILTLLKQFASHVIPV
SVSGLLEGIGNIGISLVNSFTSLPGGLPTGITIPTGTVKLANLDEAFVFVAKLLETYPAAVKDLSPVQISAFLSGKGGLEAVLSNIVAF
LTDLLKHPAHLKSLNIPLSPESINKLESALVLLRASIIDVASPEISRKFTTQIDEILTLLKQFASHVIPVSVSGLLEGIGNIGISLVNS
FTSLPGGLPTGITIPTGTVKLANLDEAFVFVAKLLETYPAAVKDLIPVQISAFLSGKGGLEAVLSNIVAFLTDLLKHPAHLKSLNIPLS
PESINKLESAFVLLRASIVEVASPEISRKFTTQIDEILTLLKQFASHVIPVSVSGLLEGIGNIGISLVNSFTSLPGGLPTGITIPTSTV
KLANLDEAFVFVAKLLETYPAAVKDLSPVQISAFLSGKGGLEAVLSNIVAFLTDLLKHPAHLKSLNIPLSPESINKLESALVLLRASIV
EVASPEISRKFTTQIDEILTLLKQFASHVIPVSVSGLLEGIGNIGISLVNSFTSLPGGLPTGITIPTGTVKLANLDEAFVFVAKLLETY
PAAVKDLSPVQISAFLSGKGGLEAVLSNIVAFLTDLLKHPAHLKSLNIPLSPESINKLESALVLLRASIVEVASPEISRKFTTQIDEIL
TLLKQFASHVIPVSVSGLLEGIGNIGISLVNSFTSLPGGLPTGITIPTGTVKLANLDEAFVFVAKLLETYPAAVKDLSPVQISAFLSGK
GGLEAILPNIVEFLKELLKEPAHLKSLKTALSLESINKLESAHFLLLSFIKDKVSINVSRQIISDMNTVLELISKVKNFVYPLLDINSR
HVVNIDRPSLIENPKITAVQIRDTRRETIASNPNDFEALPLLPEIGQDVDKDSDINVEKSLEIGESVIPIINDLVSPSMNDHIAELATD
VNAFIDIFTIGNLTQIDNIIKGKVFTLPSGLILSLPALLDKILSATSLSNCISNSRLLSLNEILGKLLIIASKLPQNLDSKDIIDKISV
TKNVVNVINIQTESLTINELQKILSAIETSILSQNPKMLNVIINEQTFLGPMGLRLSFKDVLKYIAFIPTHKQNLNAVQLQTLNLTLAR
IRNLILKTPISNDTTTILKAMYSAKKTLDQMILATKENIIDKFATIVTKFTTVIRSSTVKQVFAVIKGDDELATDDLDFIVDNVAKKIS
LVDLLTMILKHSEFYGSSLAVFRLKYRLDSLQNAISTLPNTPDVQVVLSLLSKVSFAVNKLAHANNQFTIDVIIQFVSIFKKILSTGTT
LEVNNIISGHSVSGPNALTSSLAFLLEKIFYISVETKNVNPVILIELKKSLSDIQTIMIKLPETENSVDLMSKLSASDVMLENVIRQIS
HTSVSTYNDFLIKFIAIIEKGPLSRINEILSGLQITGPKGLKLSLWTTLSILSKISTDFEGISLESLSALDAVIKKLRTVLVRLPQSST
DTTELLGQILEVEKASKSVNGQLSLLNVKNILEFISILNSSLNKGISSQIALILSGEPINGPNGVRLSLQSLLNIIRSLKLTDLNSDEI
NSLQTGLIQVNIALSKQPITPLLEYNLSKLTLVINTVKTISLYLRKLSIELISQSLASFTRLLKLSASDRYIDILSRGTISIDGRVKVT
LLDITNYIAQLSAQSDGVTFVQLLALQRQLLKLQVYSKSQSNNAISTYVYQQLSNTLNHVKSLLIEIKTLDLLTLSKAAVTILTDGTAD
QIQALLKGNVYKVGEMQTTLPALMDSISNLPSAAEIETIDYRKIVEALKISLAKSAGILENMRATEAAISYTLVFNTLEKVLSLLEY

F) Cadhezin-like 3 (PV091733)

MRASICLAILLVAGVVAEEKSKTISLEKKLDKRNLFDLGYGYGVPGLDLGYIGNEGLIGGRSLLGNGAQWSNGYLNGGLSSARTYSDVL
GYGNAGWSGLNGWNANGAWSGENAVLGDGAWYGGSGAYANGALLGGRYLGNEAWGGEGAVVGGQAWLGEAKLLGAHADILKTVTLLKGV
PVPVEVRKPVPYPVEKRYPVPVKVPVPQPYEVVQRVPYSVKVPVQVPVEVPQPYTVERRVPYPVRVPVDKPYPVQVRVPQPYTVEKKVP
YPVQVPVPTPYEVVKNVPYPVQVKVPVPQPVEVIQNVPYPVQVPVNKPYPVSVPQPYPVEKKVPVPYEVRRPVPYPVRVPVDKPYPVTV
EKAVSYPVRVPVPTPVPVYNRVPYDVPKPVPYPVQVPVNKPYPVQVNRPYPVQVNKPYPVPVQVPVQIPEKPHLVVDTVRQVLKPHGGA
QIIGGGILGGAYGAGYAGGYYGGEADISGLYGYGGIKSFGSTPATLGLGESAFLGSTVSPYLAGGLSTFGSSISPYAYGGSSFLGSTAT
PFAAGWTGSSTISPYSFGSTAATLAAGEGSWLGGGSGGYSLAGSAAAALAAGEGSWLGGGNGGYSFAGSTATPFSASGASLLRSTISPY
TISGGYSLGNGYQKLSNLETSYSK

G) Cadhezin-like 4 (PV091734)

MRASICLAVLLIVAAAAEEKKTLSLEKKLDKRGLFDLGYGYGVPGLDLGYIGNEGRFGGAYLGKSSLLNGGAYLNGGAYLNGGLSSALS
YSDVLGYGKSGWSGSNAWNANGALLGENSLGLGGAWSEGIGEYGNGAWLGGKGLLRNGAVLEGDTLLGSGAALEGESLYGSGALLEGGA
LLGGHALYGNEALLGGHAYGGEALVGGGAWLGAAKLLGEHENTLKTVTVVKGVPLPVQIQRPVPVPVQRNIGVPVKVPVPQPYPVEKKI
PYPVNEYVKVPVPTPHPYPVEKQVPYPVQVPYDRPYPVKVPVAQPYPVEKRIPVPYQVQVPQPYPVEKRVPVPYQVRVPVPQPYQVIKK
VQVPYKVPVDRPVPVPVPHPYPVERRVNVPYKVDKPVPYPVQVPFDRPVPYQVVKQVPHPVQVPFPQPYPVYKQIPYGVPKPVPYPVQV
PVNRPVPVKVEHPVPYPVEKPVPYPVKVPVAISSPAPVVVDTIRAIYTGHGGANAGVYGAEGYGYGGLKSIGSTIAPLGAVESGIYGST
AAPFLGSSIASLGEGEAALYGSTASPYLAGSIYGSSAYPLGEAGWAGSSIGSGWTGSSTISPYSYGGLSSIGSGWTGSSTISPYSYGGL
TSIGSGWSGSSTISPLSVTGGYSLGQNIK

H) Zonadhesin-like A (PV091735)

MECVSKCLFAVFILNFANVAEMADPRCASALEEWSECSAHCQPTCAQPGPDIPCTRICLPGCVCQPGMVRTASRDCIRPEQCNKDSMTC
PGPNEVFDRCGANGCQNTCQKPSKSASCDSLGCSIGCICQSGYVRNGDNICTLAESCVKCDDKHARWDNAGATGCQNTCREPERSTYCR
PSGPEAGCVCVDGYLKNTTSDRCEPAESCGYCSDPNARYSLTEAHACQSTCANPDLASRCRPDGPRKGCVCRENFLRREANGCCVLACH
CRRKKI

I) Zonadhesin-likeB (PV091736)

MDYIIWQWIVAVTFLATRVESADPRCATELEVWSECGAQCQLTCAEQGPVSCEKNCVPGCVCQEGLVRTAIGRCIPAKECDENSMKCPC
PNEIYDKCGAHGCQNTCESPFLITYCDPYGCSNGCICERGYYRDLNKTCVSGEICVKCEDVNARWDNDDATMCENTCQRPDLSTYCKPS

GAEAGCVCKPGYLKDKTTGKCEPAEVCGHCDDPNAVYSLTGANSCQNTCEAPDIAPRCRAIPRAGCMCKNSDFVKNSNGLCVKLSECST
KPCGVNEHLEKNPPDCPPKVCRGIGVAYDCANVEPPQTITLKCVCDRNHLRDERSICIPVQECPAIDCNNANEVYDPCPVYSVSPGCED
LKNGLPPQNLDPNRDCFPQCRCQDGFYRSPKGACIAKEECFDCSRPNEEYRESGSVCQKTCNNLYSEKPCILAQVIDGCFCKKGFVRDS
KNNCIKPQECYKDLCGKNEIRSCGSACDTTCATLGQQCPIINIMCNDMCYCNHGYARDDCGACIPQKMCKVQGGCGGSPHARNVSCPDP
CPATCSSYSENQSTEPAPCKRLCTMYGCQCDKGFVRLESSPIDYCVDPRDCKKCTGQNEHWSRDEDCSCADLDSPRKKKSTTRKPGCRC
MDGFVRNQEGLCIQAGLCPRCGPLEVYNKYGPSCPATCRTKDKITQTMCIQSTTEGCFCKEGLLRNDDGKCVHPRECCGCDGDPNATHG
TYSPTCALTCDNYRAKLVCLDSVTEVGCSCNKGFVKDPISGLCVTTNKCLEGCGGDVNADFLPCGNDCEKTCEHPEDRPCTKMCKIGGG
CGCKAGYVKGANGRCILPSQCPKKCDDVNAVFMPCGNNCPRDCKNPGPRPCPEICKLLGECGCKPGYVEGPDKKCILPSECPNACGGDI
NASNMSYNPDCVTTCDNYKEKIECNSKAVTQGCSCNKGYIYDQIAKKCVLAKDSPGCRGDPNARFYPCTWCPQTCSRLNDQLMCIALCR
YPGNCGCNSGYVLNDKGICVRPKACPPKRCARGEKWDCPRYCRPNLCSEKDLKYKCPPQTQEQIDFCTPDCVCREGRLRDDNGNCIPED
QCPPTTQ

J) Zonadhesin-like C (PV091737)

MSLFLGYSYFVVFLLCSVRAADPRCSSTLEVWSECDAFCQKTCANPGGTQPCPESCTPGCVCGEGLVRNGSRDCIAASQCTATSMNCPG
PNEVISQCQANGCQNTCAEPGRIAYCDPYGCSFGCICKEGYLRDANEVCTWAEDCVKCEDKYARWSNVNATSCQNSCAQPDLSSYGCKP
GETGAGCVCVEGHLLGKDGTCLPAESCAFCDDPNAVYSLTLATGCYNTCENPTFETLCRPSGPKAGCICKEGFVKNSKGLCVTLKQCKT
SVCGINEHLDPCARTCPPETCASNEMRFKCAAQPPKDCKAECRCNVNHKRDARGICVPIEKCPAFDCPGQYEVYVACPSFCPGENCQDS
LSQPQCPLTIDNICQPSCRCLDGFYRNENNSYTCVPKEECFKSCSDENEEYVENGPSCQRSCNSMYLEDFCIEQVQSGCFCKKGYIRNS
RKKCIQVEDCYLDLCGTNEFRSCGSACDTTCATLNVTCPIINVQCNDMCYCVEGFARDDCGKCIPITQCPVNGGCKGDMNAKMVSQTNP
CPAKCDNSQQQPCTALAIREGCQCNKGYIQDETTNNQCVKPESCHKCNGPNEVWSRNRDCTCADITGESQISLVSASREGCRCIDGYVR
DINGICIQAGTCPRCGPLEEYKECGSACPVTCENRFQDLFCIALCVKGCFCKQGLIRDLNGKCVLLDQCPTGCGNDINASKRTFSPSCE
RTCDNRLAEKLICDETNTIEGCSCNKGYVKDTTTNLCVKPDECRAGCGGDGNAEFNACGNTCPTTCDNLGQNRPCSLLCRTEGGCVCKP
GYVQGGPFNRCILPEQCPRCGSNAFYSCGSACQTACSTLNETCPIINIRCNDGCYCSEGYARDAYGRCIPDKLCPPKVGCNGDINAYSV
KCPSALCSVTCENYKDTTEPQPCDEYCPRDGSKCLCNKGLLLDQNTGFCVKSENCNKCTGQNEVSSNGFDVTCAERDSPNFRNILRTRK
PTLGCHCKIDFIRNANNECIPAHLCPQKCGSLEEYMECGSACPATCRNLKEPNRICTDQCLVGCYCKSPLVRNDRGQCVYPDQCLSLFG
CGGDINASKKNYLVGCVPTCDNPKALENCAAVPTPQSTGCVCNEGLVYDGISKKCIPPSLCLPGCNGDPNAQFLACGNNCPVTCDNFAD
PPRCIDVCKVNAGECGCKPNFVRNSRGMCVRPSQCGEYYVTLDI

K) Zonadhesin-like D (PV091738)

MIQLEISIFLCLITTSMATVKLCSGRNEEFREGSVCQADCSNLYSDMMCISKLIQECYCKKGYVRNYKWKCIKVEDCYKDRCGTNEIRA
CGSACDTTCATLGKPCPIMNAQCVDMCYCAEGFARDDCGKCIPQADCPAYNCTGPNEEYSRNTDCTCDDLDQDACTMPPSKNAGCRCID
GYVRDKNGVCIQAGLCPRCGPSEYFTKSACPKTCENMNFEKRCKDRPKYGCFCKEGYVRAQNNMCLLPEYCSPMLDESMM

L) Mucin-like 1 (PV091739)

MATRGGQMERIAALAALLIVGKALGDSQNATATAGSPVSSREPRARSASIVGGKDFRCPSEGFHEDPNDCQVFYRCIEWGNGKYTSFRF
QCNSGTIYDPESNICNYPASTRRWQCGGKKFIAAAPSVDNNVETSKPPKKRQPSYVPVQQTNSPTTMKPSPVTTSSKPKPSYTQVFLSS
TRAPRQPEKYVDSGTSDEDKCESEGFKGDSKDCRKFYRCVGNGKGGYTRYEFSCSEATVWDQDIQGCNHPWAVKDKKCDKLHKQAQPGS
SRNADTTRYRPQVVQSQNQYNNGSDSSQSQSQENYGSSVSQQQSQQNYGSQSTQSQSQVNHGDNSNQSQTQSNSGQQSSQNQTQVNHGN
NSNQNQTQSNTGSQSSQSQSQENYGSQSSQSQLQQNQGSQSSQSQSQENYGSQSNQSQIQNNYGGQSGHNGGSQTGSSQSGGSQSSGSH
AGSSQSSQEGQGSQGSHSQKPQGGNKCTKSGFIGDSKDCKKFYRCVPNGQGGYTKYEFSCGDGTVWDQEIQSCNHAWAVKGCGGSQSTH
EQGSGQGTTESSDSNEISGESQQSGSQGEKPTQKPTPTKKPTKQPTTQPPKTKPPTTTESYKEPDTSTRPPVKPSNDKCTSEGFIGDSK
DCKRFYRCVNNGQGGFTKYEFSCGEGTVWDQDIEACNHDYAVKNRQCSSSNSGTAGSNGESVSSKPTPSTTKRPKPSSSTTKQPTTNRP
TDSTWTENPKPTSKPTSKPKPPVSGDKCTQEGFIGDSKDCRKFYRCVSNGNGGYTKYEFSCGEGTVWDKEIESCNHEWAVQKSSCGSGV
TEASDTSTQGENDKEKPSTTKRPKPSQKPTESPTSESSTDSTWTEKPKPTSKPKPSSPGNKCTQEGFIGDSSDCKKFYRCVNNGQGGYT
KYEFTCGEGTVWDTDIESCNHEWAVKKCGGSEGDNVSTSKPNTTSKPSTNPTVDSRPTGTTYTPTEATKVTKKPTSDGSCSSEGFHPDP
NDCKNFYRCVDNGKGGYTKYDFSCGDGTVWDPEIEACNHEWAVKDCGKDKGHGTTSKYEEPQTKPTSRPKPQPTEAPEYTEGYTESSTS
RRPPTSNTGKCEREGFYGDSNDCKKFYRCVDNGRGGYTRYEFSCGEGTVWDQEIEGCNHEWAVKNCGGSSVTQSPSYGEEDSTTKKQTQ
QPKPSTEPSTSSTSTSTSSYEEKPHKPPTNGGKCTSEGFYPNESDCKKFYRCVDNGKGGYTKYEFSCGEGTVWDQEIQGCNHEWAVKKC
GGSSSPSETPIQSTYSPEPEVTPTPEKPPTQSSEKPPAQGSERPNKPPSTNGKCENEGFYGDPDDCKKFYRCVDNGKGGFTKYDFSCGE
GTVWDQEITACNHPWSVTNAVCEGGSPQPQPKPETEPESTTASQKPAPQTTTERKTTTTTKRPTSERPCSQGSTTSTTTTTEEPPSSCD
GGKKDDKTTVSCPHEGFYAHPNDCKKFYRCVDWDGEGKRYSVYFFDCPEGTIWDPAVTTCNHEESVYPPRDCGQRSPSEGSSESTTSKQ
TSAPPCKTNETTTEEPTTTTTEKTTTEESTTDSTTTTTESTTTTTEGTTTTEGTTTTEGTTTTESTTTTTEATTTTEGTTTTTTEATTT
TTESTTTEATTTTESTTTEGKTTTESSTTTEGSTTTEGTTTTESSTTTEGSTTTEGTTTTEGTTTTEGTTTTEGSTTTEGSTTTEGTTT
TEGTTTTDQPTTQESTTQESTTEESSTTESSETTTEGSSDDDKCPETEDDQELYVCPTGFRRHPKYCNLFYQCTSNEDNYDMKIAVFQC
PNNTIYDQKNTQCLSENKTDQKCKGEIAQQSSYRRLDDNSRPPMTMRSKRSLCPTEGHFAFDKSDRCSNAFIKCRQSDSSASMRGFVYQ
CPKGYIYWPVSRRCERSSRVSGCDRADDNWERRWDIPVETLNVAI

M) Mucin-like 2 (PV091740)

MTKLPHRTVGITPLVYAMALTSLCIQTSAVSFPRNIQEVKDSARSDDIRSFNVAQASQYSASFSSGSSKTSAETIGNAFADIDLQKLPG
QVHSSSKVVGCTDPGGCAGLTVEDGRITDRYGDQSILSGAPVDEATGGKQFAFSQNGAENAELPANGPFWWMKADSPFRNAYQAYLKFK
STGIAEPPSVCSSNGCTFSGPESSATAPSVHIEGKPADIKNNPYINGQFEALAESYLKSQGSSRNEGALKIDISNNPFLNGQISGAGSS
RYESQYSGGSQYDQSQYQSSENSGSLGGSTAYGSGGSVGESAISGSAISASGVSGSGVEVQQRGSSSSSSSSSGSEEIDGGFDDQQQQI

EIDQQSIEQSVEENINTGELTVTCSGKNRVCVPKWQCLNGVVTAGNVRSAGGSKKCRASSEVCCKIANKIEGAFSSKVGSESSNSFGII
NVQGEAETPYQEIVPPQESTNNIEQSIYTQTGSTYRPGSEPGQKPGIPYLPPDTEGSLLLDETRTTKPPTQSTVKYVPVKTKPTSKPYV
PVKTKPVTPRPYSPPRATLPPYIPPKVSRPKVTPAPYIPPKRPTKGPDYLPPNAITSPRPTYKTGSILLDQTRTTPGIAPTPAYAGCAA
ALLCTPEEFCGADGVISTTTVFLTDYQKTFRVPITDCLIPETNQPGKCCRDSNYTDPWPTNQLGQWVPGAFGDDGHYTPNGPSKVTRPR
PIVTPRPVVTPRPVVTPRPKPVTFVSPVTRGPTVPTLPTYIESGSNTDETTLLLEQYRENGQCGLRRPGVPQGNKARLEVDFAEIPWQA
MILLATNKSLLCGGVITRPDVVVTTASCVQGLRAGQVQVKGGEWKLGVDEEPLPFQILTTRKIILHPGYQRGSLANDVALLVLQGAFQL
ADNIFPICLPSENESVEELYSGSSNCITTGWGKKVLQTHIPNALMHAVNLDLLNEGECRQKLQAYPDRLSSYDRKSCICGQSKNAYDNV
CKVDTGSALACDNGNGHWVLRGVYSWDTGCDIQDQFAGFYKFDVEWYWHALGLRVSTKYTIYQNSPSYFEGNGLIQTQEFSKIPSFIKE
EGSIKGRRPGGINGQVSGFFQGPGVTPTIITPRPDLGLGIIRVPGEGQNQGSVNVQLQGGIIGQGRPGYVQGPGSTPRPIIVTDQTGVT
YAPQFPTLVTVPAGAQEQGNFLVQRPGSVQGQGILTQRPLGVKGQGAILTQRPGGANEQGSGFFQGSVLNGGANRGQGSGFIQSQGAIN
VPGIATEQGTYISQSQGQNTQQGGGLFQGGVSLPTVGSLQGGFSIPTVIPLPGTVTPQVPLVTQSQGTPFFNTQGVTRFPVTPTQSVGI
VQGPGIIQQQGSIQGSYGGSIQAPRPLLRPPFVKGQSSSVNGGVNYISQQDAFKNNFGQGQLSTQNNIGGQGFIQGQGFIKGQPQGFIQ
SGGSSSSQGFTSVQNQGAVSQNGGVIKQFGQVLPVGPSVSPRPLFLQGAVGGQGAGSVQGQYEYKQSIAPLVGGYQYRQEEVIPGSELE
YHYESKKIIPGVTKVIQGPTVQYQYKTEQVLPQSLASGGNVQYQYTAEQSVVPQVSGGSYKVSYQSDLSQPKPQINVQYQTKEFGKPSN
SGSYTIDASGSGLLPTIVTPGVSQFGIKDAYALNNEAQASDVDCECLDDYA

Table S1 – Protein composition of the *N. cornuta* cocoon. Cocoon proteins were dissolved in 8 M urea and digested with trypsin. The resulting peptides were identified using peptide mass fingerprinting by comparing their experimental and predicted MS/MS spectra. The table shows the results generated by the MaxQuant software. For each protein, the table contains the contig identifier, GenBank accession number, intensity, and number of peptides associated with each protein. Color coding distinguishes the protein groups: yellow for structural proteins, gray for enzymes, blue for conserved proteins with known functions unrelated to silk structure, and green for proteins with unknown function.

Protein name	Contig number	GenBank	Intensity	# pep.
sericin-like prot 3,	JAHKQU010000031.1	PV091730	1055400000	15
zonadhesin-like C	JAHKQU010000021.1	PV091737	78565000	17
zonadhesin like A	JAHKQU010000021.1	PV091735	77708000	9
cadhezin- like 2	JAHKQU010000021.1	PV091732	57664000	11
sericin- like 2	JAHKQU010000031.1	PV091729	49664000	7
sericin - like 1	JAHKQU010000031.1	PV091728	24196000	5
mucin-like 1	JAHKQU010000032.1	PV091739	23805000	12
zonadhesin-like D	JAHKQU010000021.1	PV091738	19711000	3
zonadhesin-like B	JAHKQU010000021.1	PV091736	10608000	6
mucin-like 2	JAHKQU010000010.1	PV091740	4700000	3
cadhezin- like 1	JAHKQU010000021.1	PV091731	4655500	7
cadhezin- like 3	JAHKQU0100000101.1	PV091733	90591000	14
cadhezin- like 4	JAHKQU0100000101.1	PV091734	51602000	11
myrosinase 1-like	JAHKQU010000023.1	PV091742	489450000	19
serine protease, rich in cysteine	JAHKQU010000017.1	PV091743	393030000	9
inositol phosphatase 1, multicopy	JAHKQU010000025.1	PV091744	171070000	8
glucose dehydrogenase [FAD, quinone]-like	JAHKQU010000026.1	PV091745	91242000	15
juvenile hormome esterase	JAHKQU010000026.1	PV091746	65912000	14
uridine nucleosidase 2A	JAHKQU01000001.1	PV091747	64140000	9
heme peroxidase - like	JAHKQU010000017.1	PV091748	53079000	13
laccase 2 (lac2), cuticle tanning	JAHKQU010000024.1	PV091749	48446000	4
calphotin-like	JAHKQU010000019.1	PV091750	38854000	5
antichymotrypsin-2-like	JAHKQU01000001.1	PV091751	24582000	5
uridine nucleosidase 2B	JAHKQU01000001.1	PV091752	17826000	4
superoxide dismutase_TB-SOD	JAHKQU01000004.1	PV091753	16148000	7
C-type lectin 4 (CTL4)	JAHKQU010000012.1	PV091754	9424100	3
neutral ceramidase	JAHKQU01000006.1	PV091755	8334600	9
Integrase (transposon?) 2-3 copies	JAHKQU0100000100.1	PV091756	6329300	1
chitin binding, GM-P41 - like	JAHKQU010000021.1	PV091757	5531300	3
prophenoloxidase activating proteinase 1	JAHKQU01000006.1	PV091758	4289400	2
uridine nucleosidase 3	JAHKQU01000001.1	PV091759	4058700	2
chymotrypsin-1-like	JAHKQU010000026.1	PV091760	4040900	2
lysozyme-like	JAHKQU010000030.1	PV091761	2768900	1

chitin-binding domain protein 2	JAHKQU010000022.1	PV091762	2737700	2
protease with multiple trypsin domains	JAHKQU010000015.1	PV091763	1690800	2
sphingomyelin phosphodiesterase 1	JAHKQU0100000101.1	PV091764	0	1
pancreatic lipase-related protein 2-like	JAHKQU010000019.1	PV091765	0	1
cuticular protein 30 - like	JAHKQU010000026.1	PV091766	102730000	5
cuticular protein 21 - likeA	JAHKQU010000028.1	PV091767	79537000	7
cuticular protein 7 - like	JAHKQU01000001.1	PV091768	63450000	4
cuticular protein 19 - like, conserved	JAHKQU010000021.1	PV091769	26073000	2
cuticular protein 21-likeB	JAHKQU010000028.1	PV091770	14828000	6
antimicrobial 1 (Im-1-like),	JAHKQU010000031.1	PV091771	7803200	2
conserved FK506- and rapamycin-binding	JAHKQU010000018.1	PV091772	2431500	2
conserved hypoxia up-regulated protein 1	JAHKQU01000009.1	PV091773	1523800	3
possible antimicrobial 2	JAHKQU010000015.1	PV091774	1171100	1
GPS-rich, hydrophilic, unknown	JAHKQU010000031.1.1	PV091775	178400000	3
GS-rich, repetitive, unknown	JAHKQU010000022.1	PV091776	185070000	2
GNA-rich, repetitive, unknown	JAHKQU010000014.1	PV091777	126280000	1
KSG-rich, repetitive, unknown	JAHKQU010000025.1	PV091778	117540000	7
GS – rich 2, hydrophilic, unknown	JAHKQU010000014.1	PV091779	37233000	5
TPA-rich, repeats, 18 kDa, unknown	JAHKQU010000015.1	PV091780	31776000	2
SKD-rich, nenrepetitive, unknown	JAHKQU010000026.1	PV091781	31493000	5
GAE-rich, hydrophilic, unknown	JAHKQU010000028.1	PV091782	27966000	4
ILVT-rich, nonrepetitive, unknown	JAHKQU01000004.1	PV091783	27484000	3
LTA-rich, nonrepetitive, unknown	JAHKQU010000025.1	PV091784	18835000	5
unknown 11, LDI-rich, nonrepetitive	JAHKQU010000011.1	PV091785	10003000	6
SAG-rich,hydrophilic, unknown	JAHKQU010000022.1	PV091786	7207400	2
SGDY-rich, mildly negative charge, unknown	JAHKQU010000015.1	PV091787	2952700	2
TIA-rich, hydrophobic, unknown	JAHKQU0100000100.1	PV091788	2076200	1
SDV-rich, hydrophilic, unknown	JAHKQU010000031.1	PV091789	659970	1
LAS-rich, multicopy, unknown	JAHKQU0100000101.1	PV091790	249620	0

Supplementary References

1. Hall TA (1999) BioEdit: a user-friendly biological sequence alignment editor and analysis program for Windows 95/98/NT. *Nucl. Acids. Symp. Ser.* 41:95-98.
2. Kumar S, Stecher G, Li M, Knyaz C, & Tamura K (2018) MEGA X: Molecular Evolutionary Genetics Analysis across Computing Platforms. *Mol Biol Evol* 35(6):1547-1549.
3. Nguyen LT, Schmidt HA, von Haeseler A, & Minh BQ (2015) IQ-TREE: a fast and effective stochastic algorithm for estimating maximum-likelihood phylogenies. *Mol Biol Evol* 32(1):268-274.
4. Rouhova L, *et al.* (2021) Silk of the common clothes moth, Tineola bisselliella, a cosmopolitan pest belonging to the basal ditrysian moth line. *Insect Biochem Molec* 130.
5. Tyanova S, Temu T, & Cox J (2016) The MaxQuant computational platform for mass spectrometry-based shotgun proteomics. *Nat Protoc* 11(12):2301-2319.
